# New Frontiers in Potato Breeding: Tinkering with Reproductive Genes and Apomixis

**DOI:** 10.3390/biom14060614

**Published:** 2024-05-23

**Authors:** Diego Hojsgaard, Manuela Nagel, Sergio E. Feingold, Gabriela A. Massa, John E. Bradshaw

**Affiliations:** 1 Leibniz Institute of Plant Genetics and Crop Plant Research (IPK), 06466 Seeland, Germany; nagel@ipk-gatersleben.de; 2 Laboratorio de Agrobiotecnología, EEA Balcarce-IPADS (UEDD INTA–CONICET), Instituto Nacional de Tecnología Agropecuaria (INTA), Balcarce B7620, Argentina; feingold.sergio@inta.gob.ar (S.E.F.); massa.gabriela@inta.gob.ar (G.A.M.); 3 Facultad de Ciencias Agrarias, Universidad Nacional de Mar del Plata, Balcarce B7620, Argentina; 4 James Hutton Institute, Dundee DD2 5DA, UK; johnbradshaw949@btinternet.com

**Keywords:** apomixis, apomeiosis, CRISP−Cas9, endosperm development, gametogenesis, genebanks, gene editing, meiosis, parthenogenesis, *Solanum* sp.

## Abstract

Potato is the most important non-cereal crop worldwide, and, yet, genetic gains in potato have been traditionally delayed by the crop’s biology, mostly the genetic heterozygosity of autotetraploid cultivars and the intricacies of the reproductive system. Novel site-directed genetic modification techniques provide opportunities for designing climate-smart cultivars, but they also pose new possibilities (and challenges) for breeding potato. As potato species show a remarkable reproductive diversity, and their ovules have a propensity to develop apomixis-like phenotypes, tinkering with reproductive genes in potato is opening new frontiers in potato breeding. Developing diploid varieties instead of tetraploid ones has been proposed as an alternative way to fill the gap in genetic gain, that is being achieved by using gene-edited self-compatible genotypes and inbred lines to exploit hybrid seed technology. In a similar way, modulating the formation of unreduced gametes and synthesizing apomixis in diploid or tetraploid potatoes may help to reinforce the transition to a diploid hybrid crop or enhance introgression schemes and fix highly heterozygous genotypes in tetraploid varieties. In any case, the induction of apomixis-like phenotypes will shorten the time and costs of developing new varieties by allowing the multi-generational propagation through true seeds. In this review, we summarize the current knowledge on potato reproductive phenotypes and underlying genes, discuss the advantages and disadvantages of using potato’s natural variability to modulate reproductive steps during seed formation, and consider strategies to synthesize apomixis. However, before we can fully modulate the reproductive phenotypes, we need to understand the genetic basis of such diversity. Finally, we visualize an active, central role for genebanks in this endeavor by phenotyping properly genotyped genebank accessions and new introductions to provide scientists and breeders with reliable data and resources for developing innovations to exploit market opportunities.

## 1. Introduction

One of the challenges facing humanity is to maintain or improve crop yields and cropping systems while climate conditions become less predictable and more extreme. Therefore, there is a continuing need for climate-smart and disease-resistant varieties for all major crops.

*Solanum* L. is a genus with over 1500 species, including several horticultural crops. *S. tuberosum*, the potato, is the most important commercialized non-cereal crop in the world [1]. The rich history of the domestication, breeding, and transition of the potato to become one of the world’s major food crops has been considered in several studies, such as the recent book by Bradshaw [2].

Potato has a perfect multiplication system for cultivar development (sexual reproduction combined with vegetative propagation) and a long history of breeding and germplasm collection. While potato breeding has become faster and more efficient since the 1990s, linked to the development of new molecular markers and more powerful analytical tools, the genetic yield progress in potato has been very slow compared to cereal crops [2,3,4]. A consequence of this is that century-old potato varieties like ‘Russet Burbank’ and ‘Bintje’ are still cultivated. Furthermore, there is also a lack of adequate levels of disease resistance in many commercial varieties even though resistant genotypes are available in potato germplasm [3].

There are three main reasons for such a slow genetic yield gain. The first one stems from the heterozygosity of the tetraploid potato genome (AAAA, 2*n* = 4*x* = 48), sterility barriers, and inbreeding depression as a consequence of traditional breeding and selection over thousands of years. Until now, breeding for quantitative traits in potato has been carried out by sexual reproduction and intensive phenotypic selection in the resulting clonal populations, representing genotypes with unique allelic combinations. Therefore, unfavorable alleles, which have remained masked in the tetraploid genome, manifest themselves and delay genetic gain at each breeding cycle. Moreover, genetic markers associated with quantitative genes are commonly absent [5]. The second reason is related to the conservatism of potato consumers who like old familiar varieties for use as vegetables and for processing [2]. The last reason is associated with the current gaps between actual and potential yields around the globe. As potatoes were taken to different countries and selected to tuber in different climates, they reached potential yield plateaus [2]. The potential yield is the estimated yield that the crop would produce when none of the abiotic factors that define production is limited during growth [6,7]. However, despite farmers’ efforts, the actual yields in most countries lag behind the potential yields. Hence, in the short term, reducing this yield gap appears easier than increasing the yield potential.

While the consumer´s choice is influenced by economic and societal factors, the other two reasons for the slow genetic yield gain could be modulated through breeding. Until recently, inducing specific genetic changes in a target plant was difficult using conventional breeding and traditional methods of genetic modification (such as mutation breeding; [8]). However, new technologies that allow site-directed genetic modifications offer an enormous potential to bridge this gap between potential and actual yields. These techniques provide an incremental improvement, enabling genetic gains in established varieties. For example, the generation of single or multiple gene-edited genotypes tailored to consumer or industry standards could produce a ‘Russet Burbank’ resistant to late blight and viruses and a ‘Bintje’ resistant to late blight and common scab, and, thus, drive yield progress.

Particularly interesting to breeders are genes that modify reproductive phenotypes, as they expand the available breeding units or genotypes and provide new alternatives for exploiting the diversity found in germplasm in the wild and in *ex situ* collections. Traditionally, the use of natural or mutant plants exhibiting alternative reproductive phenotypes for targeted gene transfer or base broadening [9] have played an important role in potato breeding. For instance, the exploitation of unreduced (2*n*) gametes has been central in plant breeding for transferring genetic diversity to cultivated forms in many crops, including potatoes [10]. Ploidy manipulations (mostly through 2*n* gametes) have been used to overcome barriers to hybridization in interploidy crosses and to transfer genes from wild potatoes to the cultivated tetraploid gene pool [11,12,13]. These approaches rely on species that naturally display the exploited trait (e.g., 2*n* gametes) rather than genotypes genetically engineered to develop a target phenotype. Although outcrossing schemes based on ploidy bridging can be designed between species that naturally produce 2*n* gametes [13], heterozygosity transmitted to offspring is usually less than 100% [14] and outcrossing barriers often leave out many species or invoke time and resource constraints, without necessarily improving yields. For example, the introgression of genes for resistance to bacterial wilt (*Ralstonia solanacearum*) from the wild relative *S. commersonii* Poir. into commercial potato varieties has produced progenies with a wide range of resistance, aneuploid number of chromosomes, and lower yields among third-level backcross (BC3) individuals compared to the female parent [15]. Other examples of reproductive phenotypes exploited for breeding purposes, despite little knowledge about their genetic basis, are meiotic mutants, male sterile plants, and self-compatible cytotypes (see, for example, [8,14]). In most (or all) cases, the use of these reproductive alternatives has relied on plant genotypes naturally displaying these traits.

With the new genetic modification technologies, genome resources, and analytical tools [16,17], mining into the sequences of genes underlying specific reproductive phenotypes for targeted breeding is a flourishing research area in potato. This is shifting traditional breeding schemes from being based on species naturally carrying a particular trait of interest to schemes targeting any species of interest for *de novo* trait induction. A good example of the deployment of such technology and resources is the manipulation of genes determining self-incompatibility in diploid potatoes. The identification [18,19], cloning [20,21], and use of the *S*-locus inhibitor (*Sli*) gene allowed the breakdown of self-incompatibility and the selection of inbred lines for producing and trialing F_1_ hybrids. Similar case studies involving alleles from the two *S*-locus genes, the *S*-locus *RNase* gene expressed in the style and the *S*-locus *SLF* (*S-*locus F-box) gene expressed in the pollen [22,23], show that gene-editing technologies (particularly the CRISPR−Cas9 system) can knock-out *S*-*RNase* alleles and overcome self-incompatibility in diploid potatoes.

A still unexplored but appealing topic of research in potato plant reproductive biology uses multiple mutants to induce a phenotype able to mimic apomixis, i.e., the formation of clonal botanical seeds. Because plant breeding seeks to maximize genetic gain and exploit non-additive as well as additive genetic variance to improve yield [10], heterozygosity is likely to play an important role in all crops (including highly heterozygous tetraploid potato plants). Hence, maintaining heterozygosity levels in potatoes is crucial for a healthy crop and to prevent yield losses through inbreeding depression [24]. Even though sexual reproduction is a much-needed tool in breeding, it also uncouples high-yielding gene combinations and increases homozygosity, which poses challenges to all breeding programs and to the maintenance of the required phenotypic homogeneity in any cultivar, whether a diploid F_1_ hybrid or tetraploid cultivar. Hence, the use of apomixis, to freeze the genetic make-up of the plant in a clonal (botanical) seed, is a breeding technology of enormous advantages [25] and definitively a topic underexplored in potato.

About 1500 natural species exploit the complex process of apomixis [26]. It involves changes in reproductive genes at multiple stages and affects several reproductive traits. Ovules of apomictic plants can develop extra somatic embryos which replace the zygotic embryo in the seed (sporophytic apomixis) or a gametophyte bypassing meiosis (apomeiosis) and fertilization (parthenogenesis) can create seeds carrying clonal embryos (gametophytic apomixis) [27]. Apomeiosis includes alternative, species–specific developmental pathways, which, in all cases, converge in the formation of unreduced female gametophytes, whose egg-cells develop by parthenogenesis (without fertilization), but the central cell may or may not require fertilization to develop the endosperm and, consequently, an intact seed. Moreover, apomixis in plants occurs almost exclusively in polyploids, and its emergence triggers the breakdown of self-incompatibility systems [28]. Although it depends on the species, apomixis usually shows variable expressivity between individuals, and complete penetrance in natural populations [29,30]. While much is known about developmental pathways and reproductive changes, the mechanism underlying the emergence of apomixis is complex and still unknown, but is expected to involve several genes that integrate biological pathways and networks [31]. Genes underlying apomixis have only recently been found [32,33], but reproductive genes from sexual model plants that generate apomixis-like mutant phenotypes (see Section 5) have been known for a while (reviewed in [34]).

Mimicking apomixis in potato would provide multiple benefits (see below), including an alternative to sexuality for seed production, that would avoid costly hand-pollinations during the formation of diploid F_1_ hybrids for propagation through true potato seeds (TPS), and would make tetraploid propagation by TPS feasible without losing high-yield gene combinations.

Tinkering with reproductive genes to create potato genotypes with new phenotypic traits that modify biological pathways towards the induction of synthetic apomixis will provide novel plant phenotypes useful for breeding. For instance, phenotypes that bypass meiotic segregation and produce fully non-recombinant 2*n* gametes, or that blur developmental constraints imposed by the endosperm balance number (EBN), or the interploidy blockade, could allow us to open a *menu à la carte* of distinct potato wild species and landraces to be targeted in breeding programs. This article provides an overview of challenges imposed by the reproductive biology of potato species for the exploitation of potato diversity, current technological advances, and advantages concerning its reproductive manipulation. Possible target genes and available resources required for the induction of synthetic apomixis in potato are discussed, pointing out conceivable spin-off mutant phenotypes that could serve to expand breeding tools, as well as existing potato genetic resources. We showcase new frontiers of the reproductive manipulation of potato that go beyond sexual barriers towards the use of true botanical seeds.

## 2. The Needs: True (Clonal) Seeds and a Reproductively More Flexible Potato Crop

In principle, the plant material to produce potato tubers can be of various sources. Commercially and traditionally most relevant is the propagation via sprouting normal-sized (ca. 50 g) seed tubers. Alternatively, plants may develop from mini-tubers (ca. 15 g) derived from micro-plants through in vitro axillary bud proliferation or from botanical seeds, either produced by normal sexual reproduction or, in future, by apomixis. Thus, the planting material traces back to either a micro-plant or a botanical seed. The closer the planting material is, in clonal generations, to the micro-plant or botanical seed, the more likely it is to be free of disease. Which planting material is best for farmers is still an open question. However, while there is no clear answer to this question, and preferences may manifest depending on local conditions, farmer economics, and technological facilities, propagators should focus on securing and delivering cost-effective, clean (‘disease-free’) planting material to farmers [24]. For the propagation from seed tubers, this is best achieved through statutory seed certification schemes operating in areas where potatoes are grown only for seed. However, potato growing in low-income countries is characterized by informal seed systems with little use of certified seed [35], and in these circumstances propagation from botanical seed (TPS) is an attractive proposition.

The disadvantages of propagation from seed tubers are those related to the higher costs of handling large amounts of planting material (in terms of weight and size), including micro-plants (lab-produced) and mini-tubers (nursery) [36], and, in the case of new varieties, the time required to generate enough propagules for planting. Furthermore, tubers are more likely to be infected by, and to transfer, tuber-borne diseases; and their physiological age and condition determine the planting time [2]. Such challenges can easily be overcome using propagation by TPS. Potatoes develop desiccation-tolerant botanical seeds that are easily transported and stored. Seeds of wild potato species are long-lived with >96% germination after 26 years of cold storage [37], whereas seeds of cultivated species are predicted to have a medium-term storage period with 50% germination after 22 years [38] and a higher dormancy level [39]. With a few exceptions, TPS do not carry viral, fungal or bacterial pathogens, and planting time is flexible. In practice, TPS are established and particularly useful for small-scale farmers in many tropical and subtropical lowland regions around the world [40,41].

The disadvantages of TPS propagation for tetraploid potatoes, and the reason it is not adopted by large-scale farmers, agribusinesses, and the industry, is the lack of genetic uniformity in the ripening time and tuber traits that affect the yield and quality, and, in some cases, the insufficient amounts of well-developed seeds. This is because TPS varieties come from crossing heterozygous parent plants where the segregation and recombination of genes creates variation. When TPS are produced via diploid parents, including the induction of tetraploid F_1_ hybrids, lower yields might be expected compared to tetraploid potatoes that have been traditionally selected for tuber yield [3]. An alternative for the future is the use of informed crossing strategies implementing genome-wide SNP and structural variant data for the targeted development of complementary heterotic pools. The concept of “heterotic haplotypes” was first described by Snowdon et al. [42], and its potential to quickly generate effective, genetically distinct heterotic pools from non-differentiated gene pools was demonstrated in winter oilseed rape by Krenzer et al. [43]. In combination with early genomic selection, such methods could help generate effective heterotic pools, maintain and even improve the exploitation of heterosis, and develop high-performing hybrids in tetraploid potatoes.

The production of diploid inbred lines, through several generations of self-pollination, that have sufficient levels of homozygosity, vigor, and fertility for F_1_ hybrid breeding, could solve the problem of genetically uniform cultivars from TPS [24,36]. However, self-pollination is often hindered by the self-incompatibility in diploid species. Furthermore, the advantages would be limited to the first TPS generation. This increases the interest of potato breeders who can make a higher profit, but farmers would need to purchase new seeds every season, as in maize and other hybrid crops.

Avoiding the genetic consequences of sexual reproduction during seed formation by inducing synthetic apomixis in potato flowers would overcome the current challenges: (1) of using TPS for propagating highly heterozygous tetraploid varieties, and (2) of hybrid breeding, such as inbreeding, self-incompatibilities, and the development and maintenance of inbred parental lines. Moreover, synthetic apomictic potato plants producing true clonal seeds might reduce the time for the commercialization of new varieties to 1–2 years, if molecular markers were used to select genotypes with fixed beneficial alleles (or lacking large-effect deleterious alleles), compared with more than a decade through classical potato breeding or the expected 3–6 years using diploid hybrid potato technology [44]. Thus far, attempts to induce apomixis in other crops such as rice have shown steady progress, and apomictic plant genotypes with a high-enough trait penetrance for commercialization are already available (see, for example, [45]). In potato, however, experimental studies to synthesize apomixis have not yet started. The induction of apomixis was seriously considered a long time ago by Hermsen ([46], p. 604) who identified the ‘elements of apomixis’ frequently present in potatoes and those missing. However, back then, very little was known about the molecular control of the trait, and the technological tools for DNA analysis were just beginning to be developed. Other researchers have experimented with, and laterally discussed the implications of, such apomictic elements (i.e., 2*n* eggs or parthenogenesis) in breeding and for the induction of apomixis [47,48].

From a genetic and ecological viewpoint, potatoes show wide plasticity, with more than 4000 varieties catalogued [49]. They grow in a wide range of climates and geographic regions [50] and can rapidly be selected for adaptation to new local environmental conditions. However, in the past, the development of adapted cultivars was not rapid. There is now a need for new varieties with a resistance to pests and diseases, that tolerate the adverse effects of temperatures and drought on tuberization, and use water and minerals more efficiently, to provide environmental benefits, as well as meet consumer demands [5]. The production of such varieties can be difficult due to reproductive barriers and the impossibility of combining the available variants into a suitable genomic background. Hence, there is a need for a reproductively more flexible potato crop, free from pollen–pistil incompatibility barriers and restraints to endosperm development.

The prospect of manipulating reproductive genes to enhance inbreeding tolerance or facilitate targeted introgression looks promising. Turning potato into a reproductively more flexible crop can be an alternative way to enhance genetic gains from existing natural variability. Different studies have shown the occurrence of variation in the reproductive steps for gamete and seed formation among potato species [51,52], and such a natural variation has already been exploited in breeding programs with some success. This suggests that these reproductive alternatives are suitable for direct genetic manipulation if the target genes can be identified, or confirmed, to provide a stable, inducible phenotype. The use of gene editing based on CRISPR−Cas9 to break down self-incompatibility or increase the frequency of unreduced gametes, among other reproductive features, can support the pre-breeding and introgression of favorable alleles into elite domesticated potato lines. Pre-breeding in crops like wheat, with relatively fewer wild relatives, are successful examples in accelerating genetic gain through field selection, genotyping, and marker-assisted breeding [53]. Gene editing targeting reproductive functions among wild potato species (like self-compatibility and the *Sli* gene; see Section 4.1) may unlock hidden genetic reservoirs for pre-breeding and trait transfer to intermediate plant materials that breeders can use in breeding to develop new varieties and to achieve a faster genetic gain.

## 3. The Genome: Sequences of Opportunities

The release of the first potato genome sequence [16] marked the advent of more powerful genome analyses. Scientists’ innovative thinking combined with the use of a unique genetic stock have allowed haplotype-resolved assemblies of diploid [54] and tetraploid potato varieties reporting a monoploid genome size of 0.775 GB and 38,214 genes [55]. Phased chromosome-scale assemblies of tetraploid cultivars are revealing the complexity of the potato genome, holding extensive allelic diversity, preferential allele expression, and structural variation with evidence of ancestral introgressions and the retention of dysfunctional and deleterious alleles [17]. On this basis, relevant aspects of the demographic and adaptive history of modern potato [56], as well as agronomically important genes [57] and gene families [58], were identified, and site-directed mutants were generated to assign functions to predicted genes [59].

In the meantime, the genome sequencing and genotyping of hundreds of wild and cultivated accessions are becoming standard strategies for the genome-wide genetic variation analysis of genetic diversity, e.g., for disease resistance genes; for the identification of genes under selection, particularly during potato domestication (e.g., those involved in tuberization or the loss of bitterness in tubers); for the shared genomic regions and loss of synteny relevant for hybrid breeding; and to provide robust phylogenetic trees to understand the evolution and expansion of potatoes [57,58,60]. These studies have permitted segregation between the entire set of genes within a species (i.e., the so-called pan-genome) and those genes shared between all individuals (i.e., genes of the core genome). Pan-genomes are most relevant to capturing the complete genetic diversity (and reducing genetic bias in single-genome analysis), as well as to discovering the linkage relationships and functional effects of variants. In potatoes, the analysis of pan-genomes is assisting with the discovery of many dispensable genes associated with stress tolerance or pathogen resistance [61], and important biomolecules for disease control [62,63], as well as understanding disease outbreaks in potato [64].

Thus, as expected, access to the information of genomic variability present in potato has fostered functional genetic analyses, and newly discovered traits are being incorporated into breeding programs [65]. In addition, genome editing using CRISPR−Cas technology is already being used in potato to improve the food crop while enabling fundamental research and industrial applications [66]. These new technological advances come at a critical time, when potato breeders and technologists are required to create a range of environmentally resilient varieties adapted to vast agro-ecological zones and regions in which potatoes are produced to feed a growing population [2,24]. In this context, the modification of genes involved in reproductive development is relevant to overcoming biological barriers and enhancing and accelerating breeding progress.

## 4. The Genes: The Search for Key Changes in Reproductive Modules

**Figure 1:**
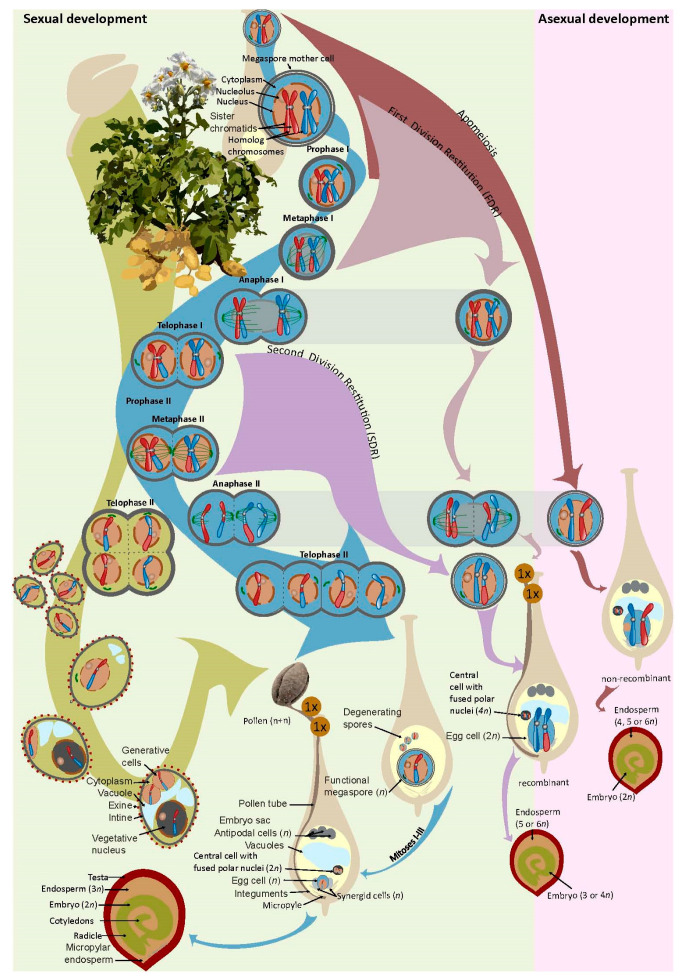
During sexual seed development, the diploid megaspore mother cell undergoes meiosis. Briefly, after disintegration of the nuclear envelope and condensation of chromosomes, homologs pair and exchange DNA segments (crossing-overs, in prophase I). Then, the bivalents attach to the spindle and align at the equatorial plate (metaphase I), and the homologous chromosomes move to opposite poles (anaphase I). At the end, spindle fibers disintegrate, and two nuclear envelopes are rebuilt (telophase I) while a cell wall is formed in between to generate two non-identical haploid daughter cells (carrying sister chromatids). Meiosis II progresses through a brief interphase and prophase II (without a DNA synthesis phase). The chromosomes attached to the spindle (metaphase II) segregate sister chromatids to the poles (anaphase II), the nuclear envelope is rebuilt (telophase II), and a cell wall is formed that generates four genetically recombinant haploid spores. In the female side, three of these spores (called megaspores) degenerate by apoptosis and the surviving megaspore undergoes three mitotic divisions (mitosis I–III) that produce a seven-celled/eight-nucleate gametophyte (Polygonum type) carrying the female gametes, i.e., the central cell with a homodiploid nuclei, and the egg cell. In the male side, the gametophyte develops synchronously and each of the four spores of meiosis (called microspores) divides through an asymmetrical mitosis that forms one large vegetative cell and a small generative cell that undergoes a second mitosis to generate two sperms. During sexual reproduction, the sperm cells are delivered, one to the egg cell and another one to the central cell, during fertilization and form a seed with a diploid recombinant embryo and a triploid endosperm. Failures in the first and second division restitution (FDR and SDR) result in the formation of un-reduced gametes with variable levels of genetic recombination and the formation of polyploid offspring. During asexual seed formation, the main meiosis steps of recombination and ploidy reduction are bypassed during apomeiosis, and consequently the female gametophyte has the same ploidy and genetic constitution as the mother plant. Clonal embryos develop from the unreduced egg-cell in such gametophytes through parthenogenesis, while the endosperm may develop autonomously or after fertilization of the unreduced central cell.

Reproduction is a highly regulated process that starts with the induction of flowering in a plant, and then goes through different developmental stages within the flower (Figure 1). These are the commitment of mother cells in ovules and anthers to generate haploid recombinant spores, the development of female and male gametophytes, the delivery of male gametes (sperm cells) to the female ones (egg cell and central cell), and, finally, the fusion of the haploid egg and sperm cells, marking the formation of a zygote together with a maternal-to-zygotic transition in gene regulation and the initiation of the embryonic tissues of the seed. All of the processes are highly regulated and involve many genes [67,68]. The search for genes, and, hence, the genetic material, with crucial roles in development and reproduction that could improve how the variability is reshuffled and exploited, is at the center of any breeding program.

In potato, despite a great variety of phenotypes and the identification of several genes with reproductive roles in pollen–pistil interactions, haploid development, and 2*n* gamete formation (Table 1), very little is known about their genetic background and molecular functions compared to other major crops, i.e., maize (*Zea mays* L.), rice (*Oryza sativa* L.), and wheat (*Triticum aestivum* L.). The main reason for this is that potato is an autotetraploid in which functional genetic studies are difficult due to tetrasomic inheritance and, therefore, studies have mainly focused on identifying nutritional or pathogenic functions of more direct use in marketing [24]. Now, however, the links between the reproductive phenotypes of interest and their genetic basis are being studied. New technological advances have provided genome-level sequence data which are unmasking an extraordinary number of genes [55]. Furthermore, the possibility to manipulate individual genes is now leading to new advances in molecular biology and potato breeding.

### 4.1. Genes Modifying Pollen–Pistil Interaction

A gametophytic self-incompatibility (GSI) system prevents successful self-pollination in most diploid potatoes which comprise potato wild relatives, cultivated landraces, and dihaploids derived from tetraploid cultivars [82,83]. It is controlled by a multiallelic *S*-locus on chromosome 1 [84,85]. Genetically, pollen is rejected when there is a match between the single *S*-haplotype in the haploid pollen and either of the two *S*-haplotypes in the diploid pistil which are both expressed. Thus, the cross *S*_1_*S*_2_ × *S*_3_*S*_4_ produces *S*_1_*S*_3_, *S*_1_*S*_4_, *S*_2_*S*_3_, and *S*_2_*S*_4_ offspring, whereas the cross *S*_1_*S*_2_ × *S*_1_*S*_3_ produces just *S*_1_*S*_3_ and *S*_2_*S*_3_ offspring and the cross (or self) *S*_1_*S*_2_ × *S*_1_*S*_2_ produces no offspring (self-incompatible). By 2011, it was known that the *S*-locus in Solanaceae, Rosaceae, and Plantaginaceae comprised at least two tightly linked loci [86]. *S-RNase* encodes S-RNases (ribonucleases with RNA degradation activity) that determine the specificity of pollen rejection in the pistil (style) and *SLF*/*SFB* encodes *S*-locus F-box proteins that fulfil this function in pollen. At the molecular level, self-incompatibility (SI) occurs when the S-RNases inhibit (self) pollen tube growth in the style [87,88], whereas pollen compatibility requires the S-RNases to be targeted for ubiquitination through the action of F-box proteins [89,90,91,92]. In simplistic terms, a plant with genotype *S*_1_*S*_2_ will produce S_1_- and S_2_-RNase in the style that can inhibit the pollen tube growth of *S*_1_ and *S*_2_ pollen, respectively, but not pollen with other *S* alleles, of which there are many. These are able to produce F-box proteins that stop S_1_- and S_2_-RNase activity [21,90,93]. In short, functional *S*-*RNase* alleles are required in the style to inhibit the growth of incompatible pollen tubes, by targeting their RNA, and, hence, prevent fertilization. Failure to secure the berry and seed set can be due to additional fertility problems. We now know that S-RNases are helped by other components such as HT proteins [94,95,96]. The GSI system breaks down in autotetraploids, at least when two different alleles are present in the pollen [97,98]. As a result, the pollen contains two different *SLF*/*SFB* genes which results in mutual weakening or competitive interaction and the degradation of all S-RNases [90]. Hence, tetraploid *S. tuberosum* can be self-pollinated.

The advent of diploid F_1_ hybrid breeding stimulated interest in self-compatibility (SC) in potato [3]. Two different ways have been used to achieve SC in potatoes, one based on the *S-RNase* gene and the other on the *S-*locus inhibitor (*Sli*) gene, as reviewed by Kardile et al. [99]. The first way either introduces dysfunctional *S-RNase* alleles from wild species or produces them from functional alleles by gene editing [22,23]. Ye et al. [22], for example, created self-compatible diploid potatoes by using the CRISPR−Cas9 system to knock out conserved regions of the *S-RNase* gene (loss-of-function) in a *S. tuberosum* Group Phureja clone. They were also able to produce selfed families containing self-compatible plants that lacked the Cas9 cassette and, hence, any exogenous DNA. The second way of achieving self-compatibility (SC) uses the *Sli* gene which was first discovered in self-fertile plants of the wild diploid (EBN = 2) potato species *Solanum chacoense* Bitt. [100], and, subsequently, mapped to the distal end of chromosome 12 [18,19,21]. A further understanding of the SC is emerging now that the candidate gene for *Sli* has been found, shown to be specifically expressed in the pollen, and to have a 533 bp transposon inserted at its promoter [20,21]. Furthermore, it has been shown that *Sli* functions like *SLF* to produce an F-box protein, PP2-B10, that destroys S-RNases during pollen tube development [21]. Hence, a name change to *non-S-locus* F-box protein (NSF) has been suggested [21]. Kardile et al. [99] mention in their review that SC could be achieved by the addition to SI genotypes of an extra *SLF* gene that can degrade all S-RNases.

The GSI system is also associated with the prevention of interspecific pollinations [94,101]. The growth of both self and interspecific pollen tubes is inhibited in the style, thus preventing fertilization. Consequently, interspecific crosses often display unilateral incompatibility (UI) in which a female SI parent prevents both self-pollination and cross-pollination by a self-compatible (SC) species, whereas a female SC parent allows cross-pollination by a SI species as well as self-pollination. Fertilization in SC × SI but not in SI × SC hybridizations was a rule proposed by Lewis and Crowe [102] that has proven to be an oversimplification because genetic differences can occur in the gene presence and function between SC species [96]. Three examples where SC is primarily the result of dysfunctional *S-RNase* alleles are as follows: the domesticated tomato (*S. lycopersicum* L.), which is a self-compatible diploid but has wild relatives which are self-incompatible [103]; the potato wild relative *S. verrucosum* Schlechtendal, which is unusual in being a self-compatible diploid 2EBN species; and cultivated diploid potatoes, in which the *S-RNase* gene is dysfunctional as a result of gene editing.

Hermsen, back in 1977, reported that *S. verrucosum* could be successfully crossed as the female parent with three SI species, diploid *S. tuberosum* Phureja Group (2EBN), *S. pinnatisectum* Dunal (1EBN), and *S. bulbocastanum* Dunal (1EBN), but not when it was the male parent [104]. It was, therefore, seen to be of value as a bridging species for the introgression of genes for disease and pest resistance from the self-incompatible diploid 1EBN species. Much more recently, Behling and Douches [96] have pointed out that, while *S. verrucosum* lacks a functional S-RNase protein, it is not known if the alleles of the *S-RNase* gene are missing, non-functional, or inhibited in some way, or if other genes such as *HT* are involved. They investigated the crosses between *S. verrucosum* as the female parent and four 1EBN species as the male parent, *S. bulbocastanum*, *S. commersonii*, *S. jamesii* Torr., and *S. pinnatisectum*. They did not observe any significant stylar barriers to pollen tube growth, and hybrid progeny was secured with *S. bulbocastanum* and *S. commersonii*. They then repeated the experiment with independent and dual CRISPR−Cas9 knockouts of the genes *S-RNase* and *HT-B* in a diploid *S. tuberosum* clone (DRH195). The results were variable but the pollen tubes of the *S-RNase* knockouts of *S. bulbocastanum* and *S. pinnatisectum* grew further down the styles than the controls, unlike the knockouts of *HT-B* alone. However, the overall interpretation of the results was complicated because *HT-A* had not been knocked out and was functional and active in the style, and possibly providing some inhibition of the pollen tube growth. Some of these complexities may be resolved now that the genomes of both *S. commersonii* and *S. verrucosum* have been sequenced [105,106].

### 4.2. Genes Modifying the Formation of Gametes and Associated Apomixis Traits

Most potato species develop a normal male gametophyte and a female gametophyte of the Polygonum type [48], which is present in about 70% of angiosperms. Such gametophytes are genetically reduced (haploid) and recombinant (Figure 1), and they fuse during the formation of sexual potato seeds. Some potato species can develop genetically unreduced (diploid) and recombinant gametes, which has a relevant role in breeding [107,108].

The ability to induce the *de novo* formation of unreduced (2*n*) gametes in potato species that lack this capacity, or, alternatively, to increase the rates of 2*n* gamete formation in potato species displaying such an ability (most diploid species), will increase the number of wild species and resources available for breeding. As the 2*n* gamete formation shows an incomplete penetrance and variable expressivity among genotypes [14], its rate could be modulated by modifying the underlying meiotic genes. The gene-directed production of potato species producing 2*n* gametes will enhance the current use of breeding schemes and ploidy manipulation strategies (e.g., [12]). This requires both gaining novel information and exploiting the current knowledge on the molecular control of 2*n* gamete formation.

Two approaches can be envisioned here: first, targeting potato genes, either newly identified and functionally characterized, underlying mutant phenotypes or known to have a relevant role during meiosis and 2*n* gamete formation (see details in Table 1); and, second, targeting potato homologs of reproductive genes functionally described in other plant systems, and identified using a sequence-homology-based strategy. A blend of these two approaches could help breeders obtain a better understanding of the molecular basis and distribution of a certain gene variant among potatoes, and then to engineer desired phenotypes in potato species lacking 2*n* gametes or increase their expressivity in species producing 2*n* gametes.

In the first case, phenotypes, such as parallel spindle (*ps*) and premature cytokinesis/omission second division (*pc/os*), responsible for the segregation of non-sister chromatids or sister chromatids to the same nuclei during 2*n* gametes formation (FDR- and SDR-like mechanisms, respectively) have been identified. Despite these phenotypes being known for a long time [70,109] and used in potato to introgress beneficial traits into commercial varieties, it is only recently that their molecular mechanisms have started to be uncovered. Cigliano et al. [69] identified three *Parallel Spindles Like* (*PSL1-3*) loci in diploid potato based on sequence homology to the *Arabidopsis thaliana* gene *Parallel Spindle1* (*AtPS1*), known to control the spindle orientation in the second meiotic division and whose defect induces 2*n* pollen. Yet, the functional characterization of *PSL* genes in potato is awaited (but see [110]).

Less is known about the molecular control of the premature cytokinesis/omission second division phenotype in potato. In Arabidopsis, at least two essential genes for cell cycle progression show mutant phenotypes that are like those in potatoes. The genes *TARDY ASYNCHRONOUS MEIOSIS* (*TAM*, also known as *CYCA1;2*) and *OMISSION OF SECOND DIVISION* (*OSD1*) are involved in the prophase/meiosis I transition and the meiosis I/meiosis II transition, and their failure leads to the production of unreduced spores and gametes due to a premature exit from meiosis, either after prophase I or after meiosis I [111]. *TAML* or *OSDL* genes in potato have not yet been identified.

The identification and *in silico* analysis of potato genes involved in ovule and seed developments is now much easier and focused on available bioinformatic tools. Strategies used in other crops or in potato for certain gene families (like the YABBY family; [112]), can be implemented for recognizing reproductive genes in potato. They retrieve sequences from different databases and use genomic and gene analyses tools for gene identification (e.g., Phytozome, the PLAZA genome database, Uniprot).

Besides the unusual spindle orientation and defects in cytokinesis and cell cycle progression, the molecular mechanism and cytology of FDR and SDR also involve changes in chromatid cohesion, meiotic recombination, and abnormal chromosomal segregation [113,114]. From a breeder´s view, the most interesting mutants in plants are those that interfere with the process of pairing and genetic recombination during the development of 2*n* gametes (Figure 1). The more recombination is suppressed, the more heterozygosity is fixed and transmitted unaltered to the offspring. Most diploid potato clones produce 2*n* eggs through SDR (by omission of the second division) or 2*n* sperms through FDR (by parallel spindles) and transmit about 40% or 80% parental heterozygosity, respectively [72,115,116]. Therefore, breeders may prefer inducing FDR as they transmit more heterozygosity to the offspring, and clonal families derived from FDR crosses outyield those from SDR and tetrasomic inheritance [117]. Yet, compared to apomixis, these phenotypes show partial effects on the levels of meiotic recombination (see below).

Apomixis and genes producing apomeiosis-like phenotypes are already known in potato and its family. Six species within Solanaceae are recorded as apomictic [26]. Most have a type of apomixis that develops extra somatic embryos in the growing ovules, and, in one *Solanum* species, *S. nigrum* L., diplospory has been recorded [118]. Diplospory is a specific type of apomixis in which meiosis progresses through a restitutional first division in the strict sense (strict FDR; [119]), meaning that homologous chromosomes fail to pair and form the synaptonemal complex (asynapsis), thus preventing recombination (Figure 1). Unlike in apomicts, in most sexual species including *Solanum*, the mechanisms of FDR usually go through variable degrees of asynapsis (broad FDR) or desynapsis (in which homologous chromosomes pair but fail to maintain pairing), and a low proportion of crossing-overs is expected [12,120]. The main consequences of these meiotic abnormalities are a shift in the levels of transmission of parental heterozygosity to the offspring. While heterozygous diploid parents cannot transmit heterozygosity by standard *n* gametes, they transmit about 80% of their heterozygosity by (broad) FDR 2*n* gametes and 40% by SDR 2*n* gametes during the formation of tetraploid offspring [14]. In apomictic species, apomeiotic (strict) FDR 2*n* gametes can transmit 100% of the parental heterozygosity [119].

With the discovery of synaptic and desynaptic mutant phenotypes, potato researchers have tried to develop genotypes that maximize the transfer of heterozygosity from parental diploids to the tetraploid offspring [121]. The combination of synaptic 3 mutant with ‘parallel spindles’ (*sy-3*, *ps*) was used with relative success to produce FDR 2*n* gametes without crossing over (FDR-NCO) and transmitting 100% heterozygosity [122]. Because of the lack of pairing during diplotene and pachytene, the chromosomal segregation in the mutant was irregular and euploid, and balanced dyads were partially recovered by the parallel spindle mechanism [122]. In a similar way, the desynaptic 1 mutant (*ds-1*) reduces substantially the frequency of chiasma which destabilizes the balanced segregation of chromosomes, and the few viable 2*n* gametes may transfer ca. 95% of the parental heterozygosity [73,120].

Recent studies have disclosed both asynapsis and desynapsis genes in potato. The meiotic gene *DMC1* plays a central role in DNA recombination through crossing over and transformants using an RNAi construct were able to knock down *StDMC1* and substantially reduce pollen viability, plausibly through asynapsis [74]. The gene *StMSH4* is an essential component of the class I crossover pathway that causes desynapsis in potato and its mutant allele leads to either highly uniform unreduced pollen or sterility [75]. The level of transmitted heterozygosity in species forming 2*n* gametes will depend upon the rates of functional crossovers during the restitution division.

A concept for fixing and transmitting the genetic makeup of parents to offspring for TPS production using unreduced desynaptic gametes was explored many years ago by Jongedijk et al. [73], but it is only now that gene-editing technologies are making their ideas feasible. While the relevance of 2*n* gametes is already known for Solanum breeding programs, it may become important for the transmission of full heterozygosity and for fixing genotypes of interest through the development of synthetic apomixis.

## 5. Breeding Strategies and Challenges to Using Apomixis in Potato

Apomictic plants skip chromosome pairing and recombination through apomeiosis to produce unreduced gametes, and create seeds carrying clonal embryos through parthenogenesis and endosperm development. Inducing apomixis in potato, or any other clonal or hybrid crop, would be a groundbreaking technology and a major boost in breeding. The fact that potato species already show evidence of the occurrence of two major components required for apomictic reproduction, i.e., apomeiotic-like unreduced gamete formation and parthenogenesis, clearly represents an advantage. Understanding the molecular basis and control of these two mechanisms that alter sexual reproduction in potatoes will then be fundamental, and their possible manipulation would be a breakthrough, which would have substantial implications for potato breeding, genetic improvement, and innovation.

### 5.1. Synthetic Apomixis

In addition, genes responsible for meiosis progression and fertilization in model plants have been used for mimicking apomixis in sexual plants, including crops (see details below), and could be targeted in potato. Synthetic apomixis is now being induced in different crops like rice, lettuce (*Lactuca sativa* L.), and tomato (*Solanum lycopersicum*), through the combination of multiple mutants using gene-editing technologies [34]. Yet, a recurrent problem with many of these mutants is that fertility is inevitably compromised and restoring fertility to a level comparable to that of sexual/wild type plants that can be used commercially is an important issue. Despite the predicted challenges for trait penetrance and expressivity in natural (neoapomicts) and synthetic apomicts [123], substantial improvements have recently been obtained [45,124] for trait deployment in crops. The strategies used so far focus on annulling key meiotic steps to produce gametes with a somatic number of non-recombinant chromosomes (mimicking apomeiosis), and then to develop the unreduced egg cell without male fertilization (mimicking parthenogenesis). Knocking out multiple genes simultaneously using CRISPR−Cas9 has produced site-specific mutant phenotypes in different plant species without side effects, both by exploiting reproductive-cell- or tissue-specific features [125,126], and by generating specific reproductive plant phenotypes such as a male-sterile *Eucalyptus* [127] or a hybrid rice producing clonal seeds [128]. In potato, the prospect for using similar approaches to create specific reproductive phenotypes is promising.

#### 5.1.1. Mimicking Apomeiosis

Despite the progress in understanding the genetics underlying apomixis in plants, apomeiotic genes responsible for clonal gametes have not yet been identified [31,129]. Thus, the focus is on manipulating meiotic genes to engineer apomeiosis *de novo* in sexual species. Researchers have been using alternative mutants and combinations of genes that abolish sister chromatid cohesion and the recombination and segregation of homologous chromosomes during meiosis to produce unreduced spores able to develop into a gametophyte. Genes which can be mutated to generate unreduced clonal gametes include *SWITCH1/DYAD* (*SWI1*) in Arabidopsis, *AMEIOTIC* in maize responsible for maintaining cohesion complexes during the meiotic prophase [130,131], and the *nonreduction in female4* (*nrf4*) mutant of maize [132], a gene that leads to both FDR and SDR 2*n* gametes and about 30% of gametes generated through a mitosis-like division [133]. Mutants for ARGONAUTE proteins (e.g., AGO9 and AGO104) and DNA methyltransferases (e.g., DMT102 and DMT103) involved in siRNAs biogenesis, chromatin condensation, and silencing have been found to produce phenotypes resembling apomeiotic pathways (reviewed in [31]).

Alternatively, a combination of different genes has been used to create unreduced clonal gametes (reviewed in [31,134]). For example, a triple mutant *Spo11–1* + *Rec8* + *Osd1* has been used to build the so-called *MiMe* phenotype that turns meiosis into mitosis [134]. *Spo11-1* is a gene required for the initiation of meiotic recombination through double-strand brake formation (a critical step in meiosis responsible for recombination) [135]. *Rec8* encodes a component of the cohesion complex essential in plants for sister chromatids cohesion during meiosis and its failure disrupts homologous pairing and allows sister chromatid segregation [136]. *OSD1* regulates the anaphase-promoting complex/cyclosome (APC/C) and *osd1* mutants show a premature exit from meiosis that skips meiosis II [111]. Thus, the MiMe mutant abolishes the meiotic recombination during meiosis I while the separation of sister chromatids and the exit from meiosis after the first division creates a mitosis-like division resulting in cells carrying a clonal nucleus. MiMe-like mutants can be designed for any species by targeting a combination of ortholog genes with similar functions, such as *spo11-2*, *prd1*, *prd2*, *prd3*/*pair1*, *DFO*, *mTOPVIB* for meiotic recombination, or *TAM1/Cyclin CYCA1;2* for meiosis exit; see, for example, [134,137].

Meiosis genes are evolutionarily conserved at the sequence level among distant eucaryotes [138] and several of these genes have been identified and their conserved function has been evaluated in genera of divergent families (in particular, Brassicaceae and Poaceae) other than Solanaceae. Implementing such a strategy in potato would require evaluating the most suitable combination of genes, including allelic variants and paralogs, independently of the level of conservatism, and considering available genus-specific alternatives. For instance, the development of dihaploids by pseudogametic parthenogenesis after interspecific 4*x* × 2*x* crosses [14,76], or after a post-meiotic restitution mechanism that produces 2*n*-eggs [77,139], points to alternative mechanisms and genes worth exploring in the gene pool of potatoes.

#### 5.1.2. Mimicking Parthenogenesis

The identification and use of genes for parthenogenesis show a high potential for application in plant breeding methods [140]. For initiating the development of the embryo in an egg-cell without fertilization and egg-sperm fusion, two genes recently identified in apomictic plants are being used to induce parthenogenesis in sexual targets. The first one, in the grass *Cenchrus squamulatus* (Fresen.) Morrone (sub *Pennisetum squamulatum*), is the *APOSPORY SPECIFIC GENOMIC REGION–BABY BOOM–LIKE* (*PsASGR-BBML*) gene [32]. This gene is an ortholog of the *BBM* gene in the clade APETALA 2 of highly conserved transcription factors whose ectopic expression induces embryo formation in *Brassica* and Arabidopsis [141]. The regulatory element in *PsASGR-BBML* is still to be proven, although it triggers parthenogenesis at a low frequency in tetraploid tobacco, but not in diploid Arabidopsis [142,143].

The second one, in the dicot *Taraxacum officinale*, sensu lato, is the *PARTHENOGENSIS* (*PAR*) gene. *PAR* encodes a K2-2 zinc finger–EAR domain protein, with the predicted DNA-binding and transcriptional repressor activity [33]. Using CRISPR−Cas9-mediated mutagenesis, these candidate genes were knocked-out and the *LOSS OF PARTHENOGENESIS* (*LOP*) mutants, identified from an earlier deletion mapping study, were resynthesized [144]. *LOP* mutants can produce viable seed only if pollinated. A *PAR* construct carrying an Arabidopsis egg-cell-specific promoter (EC1.1::PAR) was tested in the related sexual species *Lactuca sativa* and its expression induced embryo-like structures without fertilization [33].

In addition, as apomixis involves the formation of a seed carrying a clonal embryo, irrespective of the developmental origin of such embryo, two other alternatives to parthenogenesis are possible. One implies targeting genes that are involved in the development of extra (adventitious) embryos in the ovule. Somatic embryogenesis can be induced in different plant tissues by reprogramming cells to an embryogenic pathway by stimuli (heat, hormones, and epigenetic factors) and by the modulation of transcriptional regulators such as *BBM*, *LEAFY COTYLEDON*, or somatic embryogenesis-related genes [145]. The recent identification of an *RWP* gene, carrying a miniature inverted-repeat transposable element (MITE) inserted into the promoter region controlling sporophytic apomixis in citrus (*citRWP*) [145], makes it a suitable target for gene modulation using CRISPR. The RWP-RK domain-containing (RKD) family is plant-specific and its genes are responsible for maintaining the egg-cell identity [146]. The ectopic expression of *RWP* genes promotes somatic embryogenesis [147].

The other alternative implies the use of haploid inducer genes that provoke the elimination of paternal chromosomes after egg-cell fertilization. Chromosome elimination can be induced by altered centromere-specific histone 3 (*CENH3*) [148]. Using cenh3 mutants as a male parent in crosses to MiMe plants results in significant sterility due to aneuploidy, but also in a small proportion of clonal seeds [149]. Another mutant enabling haploid induction is the phospholipase A gene *MATRILINEAL* (*MTL*) identified in maize (also known as *NOT LIKE DAD* or *PHOSPHOLIPASE A1*; [150,151,152]). Likewise, *MTL* rice and wheat orthologs were shown to induce haploid seeds [153,154]. By knocking out MiMe and *MTL* genes simultaneously in rice, scientists were able to obtain low levels of clonal seeds and synthesize *de novo* apomixis, but not without a substantial reduction in fertility [128]. Fertility gains and improvements in clonal seed efficiency have been obtained by applying a similar MiMe + *MTL* approach to a panel of different rice varieties [45].

### 5.2. Endosperm Formation

Another crucial step in the formation of a viable seed is the endosperm. The exploitation of 2*n* gametes has already produced good results in potato breeding and will continue to provide breeders alternatives to cope with market demands. However, 2*n* gametes are not the only challenge to consider in introgressive hybridization. The endosperm balance number (EBN) is also a critical, but not limiting, factor during homoploid or interploid interspecific crosses. The EBN is a dosage system used to predict endosperm function in crosses between *Solanum* species and cytotypes [81]. Each species holds an EBN defined on crossability [155], and a 2:1 maternal to paternal genome dosage is needed for normal endosperm development. While such a 2:1 genome dosage is ubiquitous for almost all sexual plants and associated with the ploidy, in *Solanum* spp., *Avena* spp., and *Ipomoea* spp. [156], the dosage is independent of the plant ploidy.

In apomictic plants, the endosperm may develop autonomously (without fertilization) or by pseudogamy after the fertilization of the central cell without the restrictions of the 2:1 parental dosage observed in sexual plants [157,158]. A recently identified *ORIGIN OF RECOGNITION COMPLEX 3* (*ORC3*) gene, with differentially expressed isogenic forms in an apomictic grass, seems to be behind the tolerance of the maternal excess genome ratio during seed development in interploidy crosses [159]. In model sexual species such as Arabidopsis, the loss-of-function mutants of the Polycomb group complex 2 (PRC2), whose wild-type alleles repress endosperm formation and seed development in the absence of fertilization, cause apomixis-like phenotypes [160]. While much information has been gathered about the EBN of each *Solanum* species due to its relevance for a proper germplasm transfer during introgressive hybridization breeding approaches [161,162], much less is known about the molecular basis.

The EBN in *Solanum* species is controlled by a few major genes with additive effects and many minor genes [163,164]. Introgressive hybridization breeding schemes often produce few or no hybrids as a result of differences in EBN, and the few generated are difficult or need several generations of backcrossing for the elimination of aneuploids. Nevertheless, potato breeders have found ways to manipulate EBN in hybridizations between *S. tuberosum* and its wild relatives. Hybridization barriers can be overcome by manipulating ploidies and halving EBN through haploidization or doubling it with colchicine or through 2*n* gametes, and using special techniques such as embryo rescue [2,12,165,166,167,168]. Despite the huge potential of manipulating and converting 1EBN genomes of wild potatoes to parity with a target species (e.g., cultivated *S. tuberosum*), and of avoiding seed size variation and developmental problems in crosses where EBN are dissimilar, breeders lack genetic-level data. Recent studies indicate the existence of complex, genome-wide genetic mechanisms that govern the effective ploidy between species within an EBN category, but, thus far, attempts to identify genetic variation for effective ploidy in intra-EBN interspecific crosses have been unsuccessful [169]. Whether *ORC3-like* orthologs occur in *Solanum* is unknown. Perhaps more challenging from a breeding perspective are reproductive problems such as male (or female) sterility associated with cytoplasm types and specific nuclear genes [170]. Diploid potato landraces (e.g., *S. tuberosum* Phureja group) and their wild relatives are not yet as well-characterized reproductively as in other major crops (see for example, [171]), offering enormous potential for better understanding the remaining conundrums of potato reproductive biology and their exploitation in biotechnological applications. Future analyses and studies focusing on evaluating the molecular basis of this dosage system, and its probable links to genomic imprinting mechanisms, will pave the way to overcoming breeding barriers in potato improvement caused by endosperm malfunction and aberrant seed development.

## 6. Genebanks—An Active, Central Role in Next-Generation Breeding

In every crop, wild species have a central role in the formation and shaping of new cultivars and landraces. In potatoes, gene flow occurs in the Andes between native landrace varieties and surrounding wild *Solanum* species, both through pollen exchange and accidental collection of tubers from wild species [12,172]. Hence, potatoes are regarded as a large, plastic gene pool [173] and the potato genome as a reservoir of genetic variability to be exploited with targeted gene editing.

Genebanks are central to the next-generation breeding revolution, not only as direct providers of genetic resources, genetic variants, and novel collections, but also as active partners by producing data on collections and pre-breeding tests. Worldwide, 89 national and 4 international/regional centers, located in 59 countries, hold a collection of 82,293 potato accessions [174]. About 20% and 23% of the collections are wild species and Andean landraces, respectively, often classified into the 235 species in the taxonomy of Hawkes [175], despite the existence of a newer taxonomy [176]. Genebanks that maintain large collections of Andean landraces and wild potato species include the International Potato Center in Peru (CIP; 2596 accessions of wild species; 4468 Andean landraces), the US Potato Genebank of the US Department of Agriculture (USDA; 4044 accessions of wild species; 1177 landraces), the N.I. Vavilov All-Russian Institute of Plant Genetic Resources in Russia (VIR; 1990 accessions of wild species; 3200 landraces), the Leibniz Institute of Plant Genetics and Crop Plant Research in Germany (IPK; 1357 accessions of wild species; 2270 landraces), and the Centre for Genetic Resource, the Netherlands (CGN; 1302 accessions of wild species; 298 landraces). Most genebanks maintain accessions of wild species as TPS and store duplicates at 4 °C, 50–65% relative humidity in the short to medium term, and at −10 to −20 °C in the long term [174]. Under these conditions, TPS are considered viable for long storage periods, i.e., 92% of the *S. demissum* Lindl. seeds, 100% of *S. hjertingii* Hawkes seeds, and >96% of *S. tuberosum* groups Andigenum and Phureja seeds germinated after more than 26 years [37]. Due to the better availability of propagules and lower phytosanitary risks, TPS can often be distributed more easily than clonal plant material.

Genebanks can provide materials for prospection analyses and the continuation of earlier studies [177]. For example, the frequency and distribution of FDR and SDR 2*n* gametes can continue to be mapped, for the former in species such as *S. tuberosum*, *S. spegazzini* Bitter, and *S. infundibuliforme* Phil. [120,178], and for the latter in species such as *S. tuberosum*, *S. chacoense*, *S. kurtzianum* Bitt. et Witt., *S. phureja* Juz. et Buk., and *S. tarijense* Hawkes [72,179]. Furthermore, Hermsen [46] suggested the creation of a polyploid complex of intermating diploid and tetraploid *Solanum* species, previously treated by mutagens, as the starting material for the large-scale testing of apomixis. Genebanks can provide information on important reproductive traits and accessions (i.e., biologic materials) to create such an experimental polyploid complex. These species might include the well-tuberizing clones from (self-incompatible) diploid cultivated species such as *S. phureja* Juz. & Bukasov, *S. stenotomum* Juz. & Bukasov, *S. ajanhuiri* Juz. & Bukasov, dihaploids from complex tetraploid hybrids, and wild species such as *S. chacoense*, *S. vernei* Bitter & Wittm., and *S. microdontum* Bitter. Among the tetraploids, Hermsen [46] suggested including known genotypes of Andigena, Neotuberosum (long-day-adapted Andigena), and Tuberosum potatoes, and selected allotetraploid wild species like *S. sucrense* Hawkes. While this germplasm could still be useful, there are other genetic resources available that are well-suited to investigating and exploiting reproductive features and might prove easier for reproductive modulation and the induction of apomixis-like phenotypes.

Globally, only 37% of wild species and 43% of the landraces are accessible and provided by large genebanks such as the CIP, USDA, IPK, and CGN. The limitations in accessibility are due to not enough or poor-quality seeds/plants, inadequate plant health status, insufficient procedures for propagation and distribution, and the lack of phytosanitary certificates or required documents. In addition, some species are present in genebanks with a limited number of accessions and represent a gap in the collection; e.g., only 98 accessions of the diploid *Solanum ajanhuiri* Juz. & Bukasov are known to be stored in a few of the 89 centers, in most genebanks with <10 accessions (Table 2). More accessions would require collecting missions that are often challenged by the financial and political situation in South American countries. Other species are represented with a higher number of accessions, but identification may be hampered by the different taxonomic systems used in the potato community. Although the classification in genebanks often follows Hawkes [175], valuable morphological descriptions are also provided by Correll [180] and Dodds [181]. However, the most recent but also most debated classification follows Spooner et al. [176]. To resolve this issue, genome sequences and genetic marker information could provide a powerful tool, as well as for selection and molecular breeding. However, thus far, genetic information about accessions is hardly available. Although some collections have been intensively genotyped, the data are not systematically provided. Nevertheless, genotyping efforts by different institutions have been summarized by Ellis et al. [182], and others are findable in the database Spud DB (http://spuddb.uga.edu/) and have proven useful for resolving phylogenetic discrepancies. A forthcoming project by the CIP genebank, aimed at genotyping a substantial number of wild and cultivated potato accessions [182], will also provide useful data for identifying duplicates, misclassified accessions, and a more consistent taxonomy among potatoes.

The current genotyping gap among genebank accessions is also associated with a much larger gap in phenotyping, including reproductive phenotypes of interest for breeding, and for the induction of apomixis or apomixis-like phenotypes. Reproductive phenotyping requires skilled scientists able to identify meiotic steps and categorize abnormalities, and, therefore, has been restricted to specific studies and plant materials. Targeting the collection of phenotypic data associated with available genebank accessions or new introductions that incorporate variability is a desirable strategy to provide breeders with reliable data to plan breeding schemes and develop innovations to exploit market opportunities.

Overall, the identification of apomixis-like phenotypes poses some difficulties that will need to be resolved, to provide breeders with phylogenetically relevant information and assistance, when deciding on alternative approaches and technologies to induce and exploit 2*n* gametes or induce a more complex reproductive phenotype such as apomixis in a target species.

## 7. The Technology: Advances and Applications

As mentioned above, tetraploid potato varieties are based on a unique, highly heterozygous combination of alleles with a very low probability of replication again by crossing parental genotypes. Thus, modern potato breeding relies on the crossing of parental lines based on their phenotypic characteristics, and the production of several thousands of F_1_ plants, followed by subsequent selection processes. The improvement of established varieties (incremental breeding) was not feasible until the advent of genetic-engineering technologies such as trans/cis genesis and, more recently, genome-editing techniques.

Potato was one of the first crops for which transgenic plants were developed. There is a variety of methods for potato genome modification via genetic transformation. While many involve integrating genes of interest into the nuclear genome, plastid transformation protocols are also available [183]. The delivery and integration of genes can be achieved by particle bombardment, protoplast transfection with polyethylene glycol, and microinjection. However, the Agrobacterium-mediated transformation is the most widely utilized approach. The use of genetic-engineering approaches has allowed the successful transfer of numerous transgenes into elite potato cultivars including pest and disease resistances, abiotic stress resistance, quality attributes for improved processing, nutrition, and appearance, and novel products for biopharming [184]. Although successful developments have been obtained for a wide range of phenotypic traits, only a few genetically modified (GM) potato varieties are commercially available. These are Innate^TM^ potatoes in the US, with a resistance to black spot bruise and lower levels of asparagine and reducing sugars (low acrylamide), and a PVY-resistant variety in Argentina. The reason for this paucity of varieties could stem from a combination of factors, such as public perception and the significant costs associated with the regulatory frameworks required to obtain GM approval [185,186].

Genome-editing technologies offer promising capabilities in genetic engineering, enabling precise modifications of targeted sequences. This technology possesses immense potential to tackle numerous concerns related to cost, time, and complex biosafety issues commonly associated with traditional transgenic strategies. Additionally, the continuously evolving CRISPR−Cas toolbox has enabled multiple applications in plants, including the knockout and knock-in of target genes, modulation of gene expression (i.e., inhibition or activation), genome base editing, and many others [187,188]. Of the several methods described for potato genome editing [186], the transfection of protoplast using ribonucleoproteins (RNPs) composed of guide RNAs and the Cas nuclease is one of the most promising tools to achieve this goal, producing a high number of edited plants without the integration of foreign DNA, a major methodical problem for trait stability, off-target mutagenesis, and governmental regulations [189].

In potatoes, substantial advancements have been realized through genome editing [190], particularly in enhancing traits related to tuber quality. Notable achievements encompass the modification of starch content [191], increased resistance to bruising (resulting in reduced enzymatic browning) [189], and enhanced tolerance to cold storage achieved through the targeted knockout of the *Vacuolar Invertase* gene (*VInv*) [192].

Today, genome editing offers a range of methodologies tailored to the nature of the genetic modification and complexity of the targeted trait. While knocking out individual genes to provide a beneficial phenotype is simple, engineering complex traits is more challenging. For instance, knockout mutants of susceptibility genes (*S*-genes) for *Phytophthora infestans* such as *StDMR6-1* and *StCHL1*, or for *Potato Virus Y* (PVY) such as viral factors P3, CI, Nib, and CP, have proven to be effective in eliciting resistance and robust tolerance responses [193,194]. In contrast, the engineering of potatoes resilient to extreme temperatures and tolerant to drought and soil salinity remains somewhat limited, as the genetic bases of these traits are complex and not all genes involved have been identified [66].

Synthesizing an apomixis-like phenotype in a sexual plant requires the concurrent use of distinct gene-editing methodologies. At least three meiotic genes must be knocked out to generate the MiMe phenotype. Then, depending upon the strategy selected to mimic parthenogenesis and trigger endospermogenesis, tissue-specific promoters, and targeted gene replacement for a gene such as *BBM*, plus the use of Cas9 to recruit demethylases that erase genomic imprinting on repressed female genes and trigger autonomous endosperm, are also required [123].

In recent years, significant advances in gene-editing technology have expanded the possibilities for editing multiple genes simultaneously, whether targeting a single complex trait or multiple distinct traits. For instance, a study by Ly et al. [192] successfully edited the *VInv* and *Asparagine Synthetase 1* (*AS1*) genes associated with acrylamide production. Massa et al. [195] simultaneously knocked out *Polyphenol Oxidases* (*StPPO*s) and *VInv* genes in cultivars Spunta and Atlantic to enhance industrial and nutritional potato quality. Similarly, Zögön et al. [196] demonstrated the simultaneous editing of six genes, resulting in conspicuous phenotype alterations for a tomato *de novo* domestication assay. These studies lay the foundation for exploring multiple combinations of edited genes that could yield complex phenotypes, such as apomixis in potato.

In all the above described applications, a critical bottleneck is the regeneration of edited plants, which is genotype-dependent. This can restrict the strategies available to introduce apomixis in a certain specific potato variety or to break down the pollen–pistil incompatibility between species. Hence, preliminary protoplast extraction and regeneration assays are recommended in order to identify a specific genotype or genotypes for genome-editing applications. Recent findings of genetic factors that enhance the regeneration rates of transgenic plants [197] may provide a biotechnological alternative to overcome genotype regeneration constraints, and pave the way for the development of genotype-independent gene-edited products.

## 8. Concluding Remarks

Even though classic breeding will continue to be a major tool in potato development, the modulation of reproductive genes through new technologies enables new breeding schemes and can provide alternatives to time-consuming and resource-intensive crossings and backcrossings. The advent of diploid F_1_ hybrids allows the production of genetically uniform cultivars for propagation by true potato seed (TPS). Combining this technology with new ones focused on editing genes of interest can allow breeders to quickly improve on existing varieties, thereby increasing the rate of genetic gain.

Synthesizing apomixis in potato may help underpin and reinforce the transition to a diploid hybrid crop with the multi-generational propagation through true seeds. Alternatively, it can help breeders to capture a highly heterozygous, genetically designed background through TPS without losing heterosis. Having an apomictic potato able to fix the genomic attributes in a seed will reduce production costs and protect the varieties from pathogens. The identification of potato genes or orthologs with known functions during meiosis and embryo development is a suitable strategy to induce changes like those observed in natural apomicts. Gene editing has been successful in inducing changes in potato reproductive traits, and novel methodologies that allow simultaneous changes in multiple genes provide an excellent framework for inducing complex traits such as apomixis. In addition, potato species show a remarkable reproductive diversity, and their ovules have a propensity to develop apomixis-like phenotypes which could be further exploited to build in an apomixis phenotype. Future efforts to understand the genetic basis of this diversity will help scientists to induce and modulate such phenotypes. Detailed phenotypic analyses of reproductive stages and cellular changes are required to identify associated genes in genomic screens that could be targeted for site-directed mutagenesis. The collection of such phenotypic data must be allied to genebanks in two ways, first, as providers of accessions to be screened for reproductive variability, and, later, as repositories of well-characterized genotypes to make them available for scientists or breeders seeking innovation and market opportunities.

## Figures and Tables

**Table 1:** Reproductive phenotypes and associated genes in potato.

Mutant	Mechanism Involved	Phenotype	Gene(s) ^1^	References ^2^
*ps*	Spindle orientation	FDR, 2*n* gametes, avr. 80% parental heterozygosity transmission	Parallel Spindles Like loci (*PSL1-3*)?	[69]
*pc*/*os*	Cell division progression	SDR, 2*n* gametes, avr. 40% parental heterozygosity transmission	unknown	[70,71]
*Sy*-*1*/*4*	Chromosome pairing	Asynapsis, reduced fertility, 2*n* gametes	unknown	[72]
*Ds*-*1*	Chromosome pairing	Desynapsis, reduced fertility, 2*n* gametes	unknown	[73]
*DMC1*	Crossing over	Asynapsis, reduced pollen viability	*StDMC1*	[74]
*MSH4*	Crossing over	Desynapsis, sterility, 2*n* gametes	*StMSH4*	[75]
*Hi* ^3^	Pseudogametic parthenogenesis	Haploid induction	unknown	[76,77]
*Ms*	Plasmon factors	Male sterility ^4^	unknown	[78]
*Rf*	Inhibits expression of *Ms* genes	Male fertility restorer	*RFL-PPR*	[79,80]
*S*-locus	Gametophytic self-incompatibility system	Self-incompatibility breakdown	*S*-*RNase*^5^, *SLF*^6^ (and *HT* modifier genes)	[22,23]
*Sli*	Self-incompatibility inhibition	Self-compatible plants	*S*-locus inhibitor	[18,19]
EBN	Genome dosage	Aberrant endosperm development	unknown	[81]

FDR: first division restitution; SDR: second division restitution; ^1^ with an identified sequence; ^2^ only representative references are listed; ^3^ a mutant name for this phenotype in potato has not been defined—here, we arbitrarily took the first initials of the phenotype (*Hi*); ^4^ indehiscence, shrivelled microspores, sporad formation, anther-style fusion, ventral-styled anthers, and thin anthers; ^5^ for female specificity; ^6^ for male specificity.

**Table 2:** Availability of selected wild potato species and taxonomic classification of species present in all 89 genebanks listed at Nagel et al. [174]. For the largest genebanks, the International Potato Center (CIP) in Peru, the US Potato Genebank of the US Department of Agriculture (USDA), the N.I. Vavilov All-Russian Institute of Plant Genetic Resources (VIR) in Russia, the Leibniz Institute of Plant Genetics and Crop Plant Research (IPK) in Germany, and the Centre for Genetic Resource, the Netherlands (CGN), numbers are provided separately. EBN, endosperm balance number.

Species Sensu Hawkes [175]	Taxonomy Accepted by Spooner et al. [176]	Ploidy	CIP	USDA	VIR	IPK	CGN	All Genebanks
*S. ajanhuiri*	*Solanum ajanhuiri* Juz. & Bukasov	2x (2EBN)	14	1	9	8		98
*S. chacoense*	*Solanum chacoense* Bitter	2x (2EBN), 3x	18	167	212	111	72	717
*S. infundibuliforme*	*Solanum infundibuliforme* Phil.	2x (2EBN)	9	127	60	4	41	262
*S. kurtzianum*	*Solanum kurtzianum* Bitter & Wittm.	2x (2EBN)	3	94	117	13	34	290
*S. microdontum*	*Solanum microdontum* Bitter	2x (2EBN), 3x	14	114	34	44	41	307
*S. phureja*	*S. tuberosum* ‘Andigenum group’ diploids	2x (2EBN)	197		88		1	350
*S. spegazzini*	*Solanum brevicaule* Bitter	2x (2EBN), 4x (4EBN), 6x (4EBN)	3	n.a.	74	57	40	195
*S. stenotomum*	*S. tuberosum* ‘Andigenum group’ diploids	2x (2EBN)	110	n.a.	108	14		454
*S. sucrense*	*Solanum brevicaule* Bitter	2x (2EBN), 4x (4EBN), 6x (4EBN)	13	n.a.	26	10	41	101
*S. tarijense*	*Solanum berthaultii* Hawkes	2x (2EBN)	18	n.a.	95	19	27	186
*S. vernei*	*Solanum vernei* Bitter & Wittm.	2x (2EBN)	10	35	36	24	22	192

n.a.: not available.

## References

[1] FAO (2023). The State of Food and Agriculture 2023. Revealing the True Cost of Food to Transform Agrifood Systems.

[2] Bradshaw J.E. (2021). Potato Breeding: Theory and Practice.

[3] Lindhout P., Meijer D., Schotte T., Hutten R.C.B., Visser R.G.F., van Eck H.J. (2011). Towards F1 Hybrid Seed Potato Breeding. Potato Res..

[4] Monneveux P., Ramírez D.A., Pino M.-T. (2013). Drought tolerance in potato (*S. tuberosum* L.): Can we learn from drought tolerance research in cereals?. Plant Sci..

[5] Bradshaw J.E., Vreugdenhil D., Bradshaw J., Gebhardt C., Govers F., Mackerron D.K.L., Taylor M.A., Ross H.A. (2007). Potato-Breeding Strategy. Potato Biology and Biotechnology.

[6] Haverkort A.J., Franke A.C., Steyn J.M., Pronk A.A., Caldiz D.O., Kooman P.L. (2015). A Robust Potato Model: LINTUL-POTATO-DSS. Potato Res..

[7] Dadrasi A., Torabi B., Rahimi A., Soltani A., Zeinali E. (2022). Modeling Potential production and yield gap of potato using modelling and GIS approaches. Ecol. Model..

[8] Bradshaw J.E. (2016). Plant Breeding: Past, Present and Future.

[9] Du M., Wang T., Lian Q., Zhang X., Xin G., Pu Y., Bryan G.J., Qi J. (2021). Developing a new model system for potato genetics by androgenesis. J. Integr. Plant Biol..

[10] Dewitte A., Van Laere K., Van Huylenbroeck J., Abdurakhmonov I. (2012). Use of 2*n* Gametes in Plant Breeding. Plant Breeding.

[11] Haynes K.G., Potts W.E. (1993). Minimizing inbreeding in tetraploids derived through sexual polyploidization. Am. Potato J..

[12] Ortiz R. (1998). Potato breeding via ploidy manipulations. Plant Breed. Rev..

[13] Carputo D., Barone A., Frusciante L. (2000). 2*n* gametes in the potato: Essential ingredients for breeding and germplasm transfer. Theor. Appl. Genet..

[14] Ortiz R., Mihovilovich E., Campos H., Ortiz O. (2020). Genetics and Cytogenetics of the Potato. The Potato Crop.

[15] Andino M., Gaiero P., González-Barrios P., Galván G., Vilaró F., Speranza P. (2022). Potato Introgressive Hybridisation Breeding for Bacterial Wilt Resistance Using *Solanum commersonii* Dun. As Donor: Genetic and Agronomic Characterisation of a Backcross 3 Progeny. Potato Res..

[16] Potato Genome Sequencing Consortium (2011). Genome sequence and analysis of the tuber crop potato. Nature.

[17] Hoopes G., Meng X., Hamilton J.P., Achakkagari S.R., de Alves F., Guesdes F., Bolger M.E., Coombs J.J., Esselink D., Kaiser N.R. (2022). Phased, chromosome scale genome assemblies of tetraploid potato reveal a complex genome, transcriptome, and predicted proteome landscape underpinning genetic diversity. Mol. Plant.

[18] Hosaka K., Hanneman R.E. (1998). Genetics of self-compatibility in a self-incompatible wild diploid potato species *Solanum chacoense*. 1. Detection of an *S-locus inhibitor* (*Sli*) gene. Euphytica.

[19] Hosaka K., Hanneman R.E. (1998). Genetics of self-compatibility in a self-incompatible wild diploid potato species *Solanum chacoense*. 2. Localization of an *S-locus inhibitor* (*Sli*) gene on the potato genome using DNA markers. Euphytica.

[20] Eggers E.-J., van der Burgt A., van Heusden S.A.W., de Vries M.E., Visser R.G.F., Bachem C.W.B., Lindhout P. (2021). Neofunctionalisation of the *Sli* gene leads to self-compatibility and facilitates precision breeding in potato. Nat. Commun..

[21] Ma L., Zhang C., Zhang B., Tang F., Li F., Liao Q., Tang D., Peng Z., Jia Y., Gao M. (2021). A non *S*-locus *F-box* gene breaks self-incompatibility in diploid potatoes. Nat. Commun..

[22] Ye M., Peng Z., Tang D., Yang Z., Li D., Xu Y., Zhang C., Huang S. (2018). Generation of self-compatible diploid potato by knockout of S-RNase. Nat. Plants.

[23] Enciso-Rodriguez F., Manrique-Carpintero N.C., Nadakuduti S.S., Buell C.R., Zarka D., Douches D. (2019). Overcoming Self-Incompatibility in Diploid Potato Using CRISPR-Cas9. Front. Plant Sci..

[24] Bradshaw J.E. (2022). Breeding Diploid F1 Hybrid Potatoes for Propagation from Botanical Seed (TPS): Comparisons with Theory and Other Crops. Plants.

[25] van Dijk P.J., Rigola D., Schauer S.E. (2016). Plant breeding: Surprisingly, less sex is better. Curr. Biol..

[26] Hojsgaard D., Pullaiah T. (2023). Apomixis in Angiosperms: Mechanisms, Occurrences, and Biotechnology.

[27] Hand M.L., Koltunow A.M. (2014). The genetic control of apomixis: Asexual seed formation. Genetics.

[28] Hörandl E. (2010). The evolution of self-fertility in apomictic plants. Sex. Plant Reprod..

[29] Hojsgaard D.H., Martínez E.J., Quarin C.L. (2013). Competition between meiotic and apomictic pathways during ovule and seed development results in clonality. New Phytol..

[30] Karunarathne P., Reutemann A.V., Schedler M., Gluecksberg A., Martinez E.J., Honfi A.I., Hojsgaard D.H. (2020). Sexual modulation in a polyploid grass: A reproductive contest between environmentally inducible sexual and genetically dominant apomictic pathways. Sci. Rep..

[31] Hojsgaard D. (2020). Apomixis Technology: Separating the Wheat from the Chaff. Genes.

[32] Conner J.A., Mookkan M., Huo H., Chae K., Ozias-Akins P. (2015). A parthenogenesis gene of apomict origin elicits embryo formation from unfertilized eggs in a sexual plant. Proc. Natl. Acad. Sci. USA.

[33] Underwood C.J., Vijverberg K., Rigola D., Okamoto S., Oplaat C., Camp R.H.M.O.D., Radoeva T., Schauer S.E., Fierens J., Jansen K. (2022). A PARTHENOGENESIS allele from apomictic dandelion can induce egg cell division without fertilization in lettuce. Nat. Genet..

[34] Mahlandt A., Singh D.K., Mercier R. (2023). Engineering apomixis in crops. Theor. Appl. Genet..

[35] Forbes G.A., Charkowski A., Andrade-Piedra J., Parker M.L., Schulte-Geldermann E., Campos H., Ortiz O. (2020). Potato seed systems. The Potato Crop: Its Agricultural, Nutritional and Social Contribution to Humankind.

[36] Lindhout P., de Vries M., ter Maat M., Ying S., Viquez-Zamora M., van Heusden S., Wang-Pruski G. (2018). Hybrid potato breeding for improved varieties. Achieving Sustainable Cultivation of Potatoes Volume 1: Breeding Improved Varieties.

[37] Towill L.E. (1983). Longevity of true seed from tuber-bearing and closely related non-tuber-bearing Solanum species. Am. Potato J..

[38] Walters C., Wheeler L.M., Grotenhuis J.M. (2005). Longevity of seeds stored in a genebank: Species characteristics. Seed Sci. Res..

[39] Bamberg J. (2018). Diurnal alternating temperature improves germination of some wild potato (*Solanum*) botanical seedlots. Am. J. Potato Res..

[40] Simmonds N.W. (1997). A review of potato propagation by means of seed, as distinct from clonal propagation by tubers. Potato Res..

[41] Chujoy E., Cabello R. (2007). The canon of potato science: The true potato seed (TPS). Potato Res..

[42] Snowdon R.J., Abbadi A., Kox T., Schmutzer T., Leckband G. (2015). Heterotic Haplotype Capture: Precision breeding for hybrid performance. Trends Plant Sci..

[43] Krenzer D., Frisch M., Beckmann K., Kox T., Flachenecker C., Abbadi A., Snowdon R., Herzoget E. (2024). Simulation-based establishment of base pools for a hybrid breeding program in winter rapeseed. Theor. Appl. Genet..

[44] Mascher M., Jayakodi M., Stein N. (2021). The reinvention of potato. Cell Res..

[45] Liu C., Wang J., Lu H., Huang Y., Yan H., Liang H., Wang C., Wang K. (2023). Engineering synthetic apomixis in different hybrid rice varieties using the Fix strategy. New Crops.

[46] Hermsen J.G.T. (1980). Breeding for apomixis in potato: Pursuing a utopian scheme. Euphytica.

[47] Taylor L.M. (1978). Variation patterns of parthenogenetic plants derived from ‘unreduced’ embryo-sacs of *Solanum tuberosum* ssp. *andigena* (Juz. et Buk.) Hawkes. Theor. Appl. Genet..

[48] Jongedijk E. (1985). The pattern of megasporogenesis and megagametogenesis in diploid *Solanum* species hybrids: Its relevance to the origin of 2*n*-eggs and the induction of apomixis. Euphytica.

[49] Hils U., Pieterse L. (2007). World Catalogue of Potato Varieties 2007.

[50] Haverkort A.J., Struik P.C. (2015). Yield levels of potato crops: Recent achievements and future prospects. Field Crops Res..

[51] Mendiburu A.O., Peloquin S.J. (1977). The significance of 2*n* gametes in potato breeding. Theor. Appl. Genet..

[52] Golmirzaie A.M., Malagamba P., Pallais N., Bradshaw J.E., Mackay G.R. (1994). Breeding potatoes based on true seed propagation. Potato Genetics.

[53] Lyzenga W.J., Pozniak C.J., Kagale S. (2021). Advanced domestication: Harnessing the precision of gene editing in crop breeding. Plant Biotechnol. J..

[54] Zhou Q., Tang D., Huang W., Yang Z., Zhang Y., Hamilton J.P., Visser R.G.F., Bachem C.W.B., Buell C.R., Zhang Z. (2020). Haplotype-resolved genome analyses of a heterozygous diploid potato. Nat. Genet..

[55] Sun H., Jiao W.-B., Krause K., Campoy J.A., Goel M., Folz-Donahue K., Kukat C., Huettel B., Schneeberger K. (2022). Chromosome-scale and haplotype-resolved genome assembly of a tetraploid potato cultivar. Nat. Genet..

[56] Gutaker R.M., Weiß C.L., Ellis D., Anglin N.L., Knapp S., Fernández-Alonso J.L., Prat S., Burbano H.A. (2019). The origins and adaptation of European potatoes reconstructed from historical genomes. Nat. Ecol. Evol..

[57] Li Y., Colleoni C., Zhang J., Liang Q., Hu Y., Ruess H., Simon R., Liu Y., Liu H., Yu G. (2018). Genomic Analyses Yield Markers for Identifying Agronomically Important Genes in Potato. Mol. Plant.

[58] Tang R., Dong H., He L., Li P., Shi Y., Yang Q., Jia X., Li X.-Q. (2022). Genome-wide identification, evolutionary and functional analyses of KFB family members in potato. BMC Plant Biol..

[59] Sharma N., Siddappa S., Malhotra N., Thakur K., Salaria N., Sood S., Bhardwaj V. (2022). Advances in potato functional genomics: Implications for crop improvement. Plant Cell Tissue Organ Cult..

[60] Zhang F., Qu L., Gu Y., Xu Z.-H., Xue H.-W. (2022). Resequencing and genome-wide association studies of autotetraploid potato. Mol. Hortic..

[61] Lin X., Jia Y., Heal R., Prokchorchik M., Sindalovskaya M., Olave-Achury A., Makechemu M., Fairhead S., Noureen A., Heo J. (2023). *Solanum americanum* genome-assisted discovery of immune receptors that detect potato late blight pathogen effectors. Nat. Genet..

[62] Pacheco-Moreno A., Stefanato F.L., Ford J.J., Trippel C., Uszkoreit S., Ferrafiat L., Grenga L., Dickens R., Kelly N., Kingdon A.D.H. (2021). Pan-genome analysis identifies intersecting roles for Pseudomonas specialized metabolites in potato pathogen inhibition. eLife.

[63] Kadiri M., Sevugapperumal N., Nallusamy S., Ragunathan J., Ganesan M.V., Alfarraj S., Ansari M.J., Sayyed R.Z., Lim H.R., Show P.L. (2023). Pan-genome analysis and molecular docking unveil the biocontrol potential of *Bacillus velezensis* VB7 against *Phytophthora infestans*. Microbiol. Res..

[64] Ge T., Jiang H., Tan E.H., Johnson S.B., Larkin R.P., Charkowski A.O., Secor G., Hao J. (2021). Pangenomic Analysis of *Dickeya dianthicola* Strains Related to the Outbreak of Blackleg and Soft Rot of Potato in the United States. Plant Dis..

[65] Feingold S.E., Massa G.A., Norero N.S., Lorenzen J. (2010). Initiatives on potato functional genetics. Am. J. Plant Sci. Biotechnol..

[66] Chincinska I.A., Miklaszewska M., Sołtys-Kalina D. (2023). Recent advances and challenges in potato improvement using CRISPR/Cas genome editing. Planta.

[67] Wijnker E., Schnittger A. (2013). Control of the meiotic cell division program in plants. Plant Reprod..

[68] Khanday I., Sundaresan V. (2021). Plant zygote development: Recent insights and applications to clonal seeds. Curr. Opin. Plant Biol..

[69] Cigliano R.A., Sanseverino W., Cremona G., Consiglio F.M., Conicella C. (2011). Evolution of Parallel Spindles Like genes in plants and highlight of unique domain architecture. BMC Evol. Biol..

[70] Mok D.W.S., Peloquin S.J. (1975). The inheritance of three mechanisms of diploandroid (2*n* pollen) formation in diploid potatoes. Heredity.

[71] Mok D.W.S., Peloquin S.J. (1975). Breeding value of 2*n* pollen (diplandroids) in tetraploid × diploid crosses in potatoes. Theor. Appl. Genet..

[72] Douches D.S., Quiros C.F. (1988). Genetic strategies to determine the mode of 2*n* egg formation in diploid potatoes. Euphytica.

[73] Jongedijk E., Ramanna M.S., Sawor Z., Hermsen J.G.T. (1991). Formation of first division restitution (FDR) 2*n*-megaspores through pseudohomotypic division in ds-1 (desynapsis) mutants of diploid potato: Routine production of tetraploid progeny from 2*x* FDR × 2*x* FDR crosses. Theor. Appl. Genet..

[74] Kumar A., Siddappa S., Bhardwaj V., Dalamu Singh B., Sharma N., Dipta B., Kumar V., Goutam U., Sood S. (2023). Generation of Asynaptic Mutants in Potato by Disrupting StDMC1 Gene Using RNA Interference Approach. Life.

[75] Clot C.R., Klein D., Koopman J., Schuit C., Engelen C.J.M., Hutten R.C.B., Brouwer M., Visser R.G.F., Juranić M., van Eck H.J. (2024). Crossover shortage in potato is caused by StMSH4 mutant alleles and leads to either highly uniform unreduced pollen or sterility. Genetics.

[76] Hougas R.W., Peloquin S.J., Ross R.W. (1958). Haploids of the common potato. J. Hered..

[77] Hermsen J.G.T., Verdenius J. (1973). Selection from *Solanum tuberosum* group Phureja of genotypes combining high frequency haploid induction with homozygosity for embryo spot. Euphytica.

[78] Grun P., Ochoa C., Capage D. (1977). Evolution of cytoplasmic factors in tetraploid cultivated potato (Solanaceae). Am. J. Bot..

[79] Iwanaga M., Ortiz R., Cipar M.S., Peloquin S.J. (1991). A restorer gene for genetic-cytoplasmic male sterility in cultivated potatoes. Am. Potato J..

[80] Anisimova I.N., Alpatieva N.V., Karabitsina Y.I., Gavrilenko T.A. (2019). Nucleotide Sequence Polymorphism in the RFL-PPR Genes of Potato. J. Genet..

[81] Johnston S.A., den Nijs T.P.M., Peloquin S.J., Hanneman R.E. (1980). The significance of genic balance to endosperm development in interspecific crosses. Theor. Appl. Genet..

[82] Cipar M.S., Peloquin S.J., Hougas R.W. (1964). Inheritance of incompatibility in hybrids between *Solanum tuberosum* haploids and diploid species. Euphytica.

[83] Dodds K.S., Hutchinson J.B. (1965). The history and relationships of cultivated potatoes. Essays in Crop Plant Evolution.

[84] Gebhardt C., Ritter E., Barone A., Debener T., Walkemeier B., Schachtschabel U., Kaufmann H., Thompson R.D., Bonierbale M.W., Ganal M.W. (1991). RFLP maps of potato and their alignment with the homoeologous tomato genome. Theor. Appl. Genet..

[85] Kaufmann H., Salamini F., Thompson R.D. (1991). Sequence variability and gene structure at the self-incompatibility locus of *Solanum tuberosum*. Mol. Gen. Genet..

[86] McClure B., Cruz-García F., Romero C. (2011). Compatibility and incompatibility in S-RNase-based systems. Ann. Bot..

[87] Lee H.S., Huang S., Kao T. (1994). S Proteins Control Rejection of Incompatible Pollen in *Petunia inflata*. Nature.

[88] Murfett J., Atherton T.L., Mou B., Gassert C.S., McClure B.A. (1994). S-RNase Expressed in Transgenic Nicotiana Causes *S*-Allele-Specific Pollen Rejection. Nature.

[89] Sijacic P., Wang X., Skirpan A.L., Wang Y., Dowd P.E., McCubbin A.G., Huang S., Kao T.-H. (2004). Identification of the Pollen Determinant of S-RNase-Mediated Self-Incompatibility. Nature.

[90] Kubo K., Entani T., Takara A., Wang N., Fields A.M., Hua Z., Toyoda M., Kawashima S., Ando T., Isogai A. (2010). Collaborative Non-Self Recognition System in S-RNase-Based Self-Incompatibility. Science.

[91] Kubo K.-I., Paape T., Hatakeyama M., Entani T., Takara A., Kajihara K., Tsukahara M., Shimizu-Inatsugi R., Shimizu K.K., Takayama S. (2015). Gene Duplication and Genetic Exchange Drive the Evolution of S-RNase-Based Self-Incompatibility in Petunia. Nat. Plants.

[92] Sun L., Williams J.S., Li S., Wu L., Khatri W.A., Stone P.G., Keebaugh M.D., Kaoa T.H. (2018). S-Locus F-Box Proteins Are Solely Responsible for S-RNase-Based Self-Incompatibility of Petunia Pollen. Plant Cell.

[93] Dzidzienyo D.K., Bryan G.J., Wilde G., Robbins T.P. (2016). Allelic Diversity of *S*-Rnase Alleles in Diploid Potato Species. Theor. Appl. Genet..

[94] Baek Y.S., Covey P.A., Petersen J.J., Chetelat R.T., McClure B., Bedinger P.A. (2015). Testing the SI × SC rule: Pollen–pistil interactions in interspecific crosses between members of the tomato clade (Solanum section Lycopersicon, Solanaceae). Am. J. Bot..

[95] Tovar-Méndez A., Lu L., McClure B. (2017). HT proteins contribute to S-RNase-independent pollen rejection in *Solanum*. Plant J..

[96] Behling W.L., Douches D.S. (2023). The effect of self-compatibility factors on interspecific compatibility in *Solanum* Section Petota. Plants.

[97] Lewis D. (1943). The physiology of incompatibility in plants. III. Autopolyploids. J. Genet..

[98] Lewis D. (1954). Comparative incompatibility in angiosperms and fungi. Adv. Genet..

[99] Kardile H.B., Yilma S., Sathuvalli V. (2022). Molecular approaches to overcome self-incompatibility. Plants.

[100] Hanneman R.E. (1985). Self fertility in *Solanum chacoense*. Am. Potato J..

[101] Bedinger P.A., Chetelat R.T., McClure B., Moyle L.C., Rose J.K., Stack S.M., Kumar A., Van Der Knaap E., Baek Y.S., Lopez-Casado G. (2011). Interspecific reproductive barriers in the tomato clade: Opportunities to decipher mechanisms of reproductive isolation. Sex. Plant Reprod..

[102] Lewis D., Crowe L. (1958). Unilateral interspecific incompatibility in flowering plants. Heredity.

[103] Tovar-Méndez A., Kumar A., Kondo K., Ashford A., Baek Y.S., Welch L., Bedinger P.A., McClure B.A. (2014). Restoring pistil-side self-incompatibility factors recapitulates an interspecific reproductive barrier between tomato species. Plant J..

[104] Hermsen J.T. General considerations on interspecific hybridization. Proceedings of the 8th Congress of Eucarpia.

[105] Aversano R., Contaldi F., Ercolano M.R., Grosso V., Iorizzo M., Tatino F., Xumerle L., Molin A.D., Avanzato C., Ferrarini A. (2015). The *Solanum commersonii* genome sequence provides insights into adaptation to stress conditions and genome evolution of wild potato relatives. Plant Cell.

[106] Hosaka A.J., Sanetomo R., Hosaka K. (2022). A *de novo* genome assembly of *Solanum verrucosum* Schlechtendal, a Mexican diploid species geographically isolated from other diploid A-genome species of potato relatives. G3.

[107] Peloquin S.J., Yerk G.L., Werner J.E., Darmo E. (1989). Potato breeding with haploids and 2*n* gametes. Genome.

[108] Ortiz R., Simon P., Jansky S., Stelly D. (2009). Ploidy manipulation of the gametophyte, endosperm and sporophyte in nature and for crop improvement: A tribute to Professor Stanley J. Peloquin (1921–2008). Ann. Bot..

[109] Fukuda Y. (1927). Cytological studies on the development of pollen-grain in different races of *Solanum tuberosum* L., with special reference to sterility. Bot. Mag. Tokyo.

[110] Paparo R., Termolino P., De Palma M., Cremona G., Consiglio M.F., Conicella C. Functional characterization of Parallel Spindles Like (PSL) genes in potato. Proceedings of the 18th Joint Meeting of the EAPR Breeding and Varietal Assessment Section and the EUCARPIA Section Potatoes.

[111] Cromer L., Heyman J., Touati S., Harashima H., Araou E., Girard C., Horlow C., Wassmann K., Schnittger A., De Veylder L. (2012). OSD1 Promotes Meiotic Progression via APC/C Inhibition and Forms a Regulatory Network with TDM and CYCA1;2/TAM. PloS Genet..

[112] Mazhar H.S.-U., Shafiq M., Ali H., Ashfaq M., Anwar A., Tabassum J., Ali Q., Jilani G., Awais M., Sahu R. (2023). Genome-Wide Identification, and In-Silico Expression Analysis of YABBY Gene Family in Response to Biotic and Abiotic Stresses in Potato (*Solanum tuberosum*). Genes.

[113] Brownfield L., Kölher C. (2011). Unreduced gamete formation in plants: Mechanisms and prospects. J. Exp. Bot..

[114] De Storme N., Geelen D. (2013). Sexual polyploidization in plants—Cytological mechanisms and molecular regulation. New Phytol..

[115] Stelly D.M., Peloquin S.J. (1986). Formation of 2N Megagametophytes in Diploid Tuber-Bearing Solanums. Am. J. Bot..

[116] Werner J.E., Peloquin S.J. (1990). Inheritance and two mechanisms of 2*n* egg formation in 2*x* potatoes. J. Hered..

[117] Peloquin S.J., Boiteux L.S., Simon P.W., Jansky S.H. (2008). A Chromosome-Specific Estimate of Transmission of Heterozygosity by 2*n* Gametes in Potato, *J*. Hered..

[118] Chochlov S.S., Zajceva M.I., Kutrijanov P.G. (1978). Vyjavlenie Apomiktichiykh Form vo Flore Cvetkovykh Rastenij SSSR. Programma, Metodika, Rezul’taty (Revelation of the Apomictic Forms in the Flora of the Angiosperms of USSR. Programs, Methods and Results).

[119] Hojsgaard D. (2018). Transient activation of apomixis in sexual neotriploids may retain genomically altered states and enhance polyploid establishment. Front. Plant Sci..

[120] Jongedijk E., Ramanna M.S. (1988). Synaptic mutants in potato, *Solanum tuberosum* L. I. Expression and identity of genes for desynapsis. Genome.

[121] Hermundstad S., Peloquin S., Jellis G., Richardson D. (1987). Breeding at the 2*x* level and sexual polyploidization. The Production of New Potato Varieties: Technological Advances.

[122] Okwuagwu C.O., Peloquin S.J. (1981). A method of transferring the intact parental genotype to the offspring via meiotic mutants. Am. Potato J..

[123] Scheben A., Hojsgaard D. (2020). Can We Use Gene-Editing to Induce Apomixis in Sexual Plants?. Genes.

[124] Vernet A., Meynard D., Lian Q., Mieulet D., Gibert O., Bissah M., Rivallan R., Autran D., Leblanc O., Meunier A.C. (2022). High-frequency synthetic apomixis in hybrid rice. Nat. Commun..

[125] Wang Z.-P., Xing H.-L., Dong L., Zhang H.-Y., Han C.-Y., Wang X.-C., Chen Q.-J. (2015). Egg cell-specific promoter-controlled CRISPR/Cas9 efficiently generates homozygous mutants for multiple target genes in Arabidopsis in a single generation. Genome Biol..

[126] Toda E., Kato N., Higashiyama T., Takashi O. (2023). Genome editing approaches using reproductive cells/tissues in flowering plants. Front. Genome Ed..

[127] Nagle M.F., Nahata S.S., Zahl B., Niño de Rivera A., Tacker X.V., Elorriaga E., Ma C., Goralogia G.S., Klocko A.L., Gordon M. (2023). Knockout of floral and meiosis genes using CRISPR/Cas9 produces male-sterility in Eucalyptus without impacts on vegetative growth. Plant Direct.

[128] Wang C., Liu Q., Shen Y., Hua Y., Wang J., Lin J., Wu M., Sun T., Cheng Z., Mercier R. (2019). Clonal seeds from hybrid rice by simultaneous genome engineering of meiosis and fertilization genes. Nat. Biotechnol..

[129] Ozias-Akins P., van Dijk P.J. (2007). Mendelian genetics of apomixis in plants. Annu. Rev. Genet..

[130] Pawlowski W.P., Wang C.-J.R., Golubovskaya I.N., Szymaniak J.M., Shi L., Hamant O., Zhu T., Harper L., Sheridan W.F., Cande W.Z. (2009). Maize AMEIOTIC1 is essential for multiple early meiotic processes and likely required for the initiation of meiosis. Proc. Natl. Acad. Sci. USA.

[131] Yang C., Hamamura Y., Sofroni K., Böwer F., Stolze S.C., Nakagami H., Schnittger A. (2019). SWITCH 1/DYAD is a WINGS APART-LIKE antagonist that maintains sister chromatid cohesion in meiosis. Nat. Commun..

[132] Fox T.W., Albertsen M.C., Williams M.E., Lawit S.J., Chamberlin M.A., Grossniklaus U., Brunner G.A., Chumak N., De Asis J.B., Pasquer F. (2016). Methods and Compositions for the Production of Unreduced, Non-Recombined Gametes and Clonal Offspring. Patent.

[133] Grossniklaus U. (2019). The Quest for Clonal Seeds: Towards Engineering Apomixis in Maize.

[134] Underwood C.J., Mercier R. (2022). Engineering Apomixis: Clonal Seeds Approaching the Fields. Annu. Rev. Plant Biol..

[135] Vrielynck N., Schneider K., Rodriguez M., Sims J., Chambon A., Hurel A., De Muyt A., Ronceret A., Krsicka O., Mézard C. (2021). Conservation and divergence of meiotic DNA double strand break forming mechanisms in *Arabidopsis thaliana*. Nucleic Acids Res..

[136] Cai X., Dong F., Edelmann R.E., Makaroff C.A. (2003). The Arabidopsis SYN1 cohesin protein is required for sister chromatid arm cohesion and homologous chromosome pairing. J. Cell Sci..

[137] Mieulet D., Jolivet S., Rivard M., Cromer L., Vernet A., Mayonove P., Pereira L., Droc G., Courtois B., Guiderdoni E. (2016). Turning rice meiosis into mitosis. Cell Res..

[138] Thangavel G., Hofstatter P.G., Mercier R., Marques A. (2023). Tracing the evolution of the plant meiotic molecular machinery. Plant Reprod..

[139] Bastiaanssen H.J.M., Berg P.M.M.M.V.D., Lindhout P., Jacobsen E., Ramanna M.S. (1998). Postmeiotic restitution in 2*n*-egg formation of diploid potato. Heredity.

[140] Vijverberg K., Ozias-Akins P., Schranz M.E. (2019). Identifying and Engineering Genes for Parthenogenesis in Plants. Front. Plant Sci..

[141] Boutilier K., Oringa R., Sharma V.K., Kieft H., Ouellet T., Zhang L., Hattori J., Liu C.-M., van Lammeren A.A.M., Miki B.L.A. (2002). Ectopic expression of *BABY BOOM* triggers a conversion from vegetative to embryonic growth. Plant Cell.

[142] Conner J.A., Podio M., Ozias-Akins P. (2017). Haploid embryo production in rice and maize induced by *PsASGR-BBML* transgenes. Plant Reprod..

[143] Zhang Z., Conner J., Guo Y., Ozias-Akins P. (2020). Haploidy in tobacco induced by *PsASGR-BBML* transgenes via parthenogenesis. Genes.

[144] Catanach A.S., Erasmuson S.K., Podivinsky E., Jordan B.R., Bicknell R. (2006). Deletion mapping of genetic regions associated with apomixis in *Hieracium*. Proc. Natl. Acad. Sci. USA.

[145] Xu Y., Jia H., Wu X., Koltunow A.M.G., Deng X., Xu X. (2021). Regulation of nucellar embryony, a mode of sporophytic apomixis in Citrus resembling somatic embryogenesis. Curr. Opin. Plant Biol..

[146] Koszegi D., Johnston A.J., Rutten T., Czihal A., Altschmied L., Kumlehn J., Wust S.E.J., Kirioukhova O., Gheyselinck J., Grossniklaus U. (2011). Members of the RKD transcription factor family induce an egg cell-like gene expression program. Plant J..

[147] Waki T., Hiki T., Watanabe R., Hashimoto T., Nakajima K. (2011). The Arabidopsis RWP-RK protein RKD4 triggers gene expression and pattern formation in early embryogenesis. Curr. Biol..

[148] Ravi M., Chan S.W.L. (2010). Haploid plants produced by centromere-mediated genome elimination. Nature.

[149] Marimuthu M.P.A., Jolivet S., Ravi M., Pereira L., Davda J.N., Cromer L., Wang L., Nogué F., Chan S.W.L., Siddiqi I. (2011). Synthetic clonal reproduction through seeds. Science.

[150] Gilles L.M., Khaled A., Laffaire J.B., Chaignon S., Gendrot G., Laplaige J., Berges H., Beydon G., Bayle V., Barret P. (2017). Loss of pollen-specific phospholipase NOT LIKE DAD triggers gynogenesis in maize. EMBO J..

[151] Kelliher T., Starr D., Richbourg L., Chintamanani S., Delzer B., Nuccio M.L., Green J., Chen Z., McCuiston J., Wang W. (2017). MATRILINEAL, a sperm-specific phospholipase, triggers maize haploid induction. Nature.

[152] Liu C., Li X., Meng D., Zhong Y., Chen C., Dong X., Xu X., Chen B., Li W., Li L. (2017). A 4-bp insertion at ZmPLA1 encoding a putative phospholipase a generates haploid induction in maize. Mol. Plant.

[153] Yao L., Zhang Y., Liu C., Liu Y., Wang Y., Liang D., Liu J., Sahoo G., Kelliher T. (2018). OsMATL mutation induces haploid seed formation in indica rice. Nat. Plants.

[154] Liu C., Zhong Y., Qi X., Chen M., Liu Z., Chen C., Tian X., Li J., Jiao Y., Wang D. (2020). Extension of the *in vivo* haploid induction system from diploid maize to hexaploid wheat. Plant Biotechnol. J..

[155] Hanneman R.E. (1999). The reproductive biology of the potato and its implication for breeding. Potato Res..

[156] Nishiyama I., Yabuno T. (1978). Interspecific cross-incompatibility due to disturbed activation of the polar nuclei by the male nucleus. Breed. Sci. (Ikushugaku Zasshi).

[157] Quarin C.L. (1999). Effect of pollen source and pollen ploidy on endosperm formation and seed set in pseudogamous apomictic *Paspalum notatum*. Sex. Plant Reprod..

[158] Talent N., Dickinson T.A. (2007). Endosperm formation in aposporous *Crataegus* (Rosaceae, Spiraeoideae, tribe Pyreae): Parallels to Ranunculaceae and Poaceae. New Phytol..

[159] Bellucci M., Cáceres M.E., Paolocci F., Vega J.M., Ortiz J.P.A., Ceccarelli M., De Marchis F., Pupilli F. (2023). ORIGIN OF RECOGNITION COMPLEX 3 controls the development of maternal excess endosperm in the Paspalum simplex agamic complex (Poaceae). J. Exp. Bot..

[160] Grossniklaus U., Paro R. (2014). Transcriptional silencing by polycomb-group proteins. Cold Spring Harb. Perspect. Biol..

[161] Hawkes J.G., Jackson M.T. (1992). Taxonomic and evolutionary implications of the Endosperm Balance Number hypothesis in potatoes. Theor. Appl. Genet..

[162] Watanabe K. (2015). Potato genetics, genomics, and applications. Breed. Sci..

[163] Ehlenfeldt M.K., Hanneman R.E. (1988). Genetic control of endosperm balance number (EBN): Three additive loci in a thresholdlike system. Theor. Appl. Genet..

[164] Bamberg J.B. (1994). Allelism of endosperm balance number (EBN) in *Solanum acaule* Bitt. and other wild potato species. Theor. Appl. Genet..

[165] Eijlander R., Stiekema W.J. (1994). Biological containment of potato (*Solanum tuberosum*): Outcrossing to the related wild species black nightshade (*Solanum nigrum*) and bittersweet (*Solanum dulcamara*). Sex. Plant Reprod..

[166] Ortiz R. (2001). The state of the use of potato genetic diversity. Broadening the Genetic Base of Crop Production.

[167] Carputo D., Barone A. (2005). Ploidy level manipulations in potato through sexual hybridization. Ann. Appl. Biol..

[168] Jansky S. (2006). Overcoming hybridization barriers in potato. Plant Breed..

[169] Behling W., Coombs J., Collins P., Douches D. (2024). An Analysis of Inter-Endosperm Balance Number Crosses with the Wild Potato *Solanum verrucosum*. Am. J. Potato Res..

[170] Hosaka K., Sanetomo R. (2020). Creation of a highly homozygous diploid potato using the *S locus inhibitor* (*Sli*) gene. Euphytica.

[171] Bohórquez-Quintero M.A., Galvis-Tarazona D.Y., Arias-Moreno D.M., Ojeda-Peréz Z.Z., Ochatt S., Rodríguez-Molano L.E. (2022). Morphological and anatomical characterization of yellow diploid potato flower for effective breeding program. Sci. Rep..

[172] Rabinowitz D., Linder C.R., Ortega R., Begazo D., Murguia H., Douches D.S., Quiros C.F. (1990). High levels of interspecific hybridization between *Solanum sparsipilum* and *S. stenotomum* in experimental plots in the Andes. Am. Potato J..

[173] Quiros C.F., Ortega R., Van Raamsdonk L., Herrera-Montoya M., Cisneros P., Schmidt E., Brush S.B. (1992). Increase of potato genetic resources in their center of diversity: The role of natural outcrossing and selection by the Andean farmer. Genet. Resour. Crop Evol..

[174] Nagel M., Dulloo E., Bissessur P., Gavrilenko T., Bamberg J., Ellis D., Giovannini P. (2022). Global Strategy for the Conservation of Potato.

[175] Hawkes J.G. (1990). The Potato: Evolution, Biodiversity & Genetic Resources.

[176] Spooner D.M., Ghislain M., Simon R., Jansky S.H., Gavrilenko T. (2014). Systematics, diversity, genetics, and evolution of wild and cultivated potatoes. Bot. Rev..

[177] Quinn A.A., Mok D.W.S., Peloquin S.J. (1974). Distribution and significance of diplandroids among the diploid Solanums. Am. Potato J..

[178] Camadro E.L., Peloquin S.J. (1980). The occurrence and frequency of 2*n* pollen in three diploid solanums from Northwest Argentina. Theor. Appl. Genet..

[179] Conicella C., Barone A., Del Giudice A., Frusciante L., Monti L.M. (1991). Cytological evidences of SDR-FDR mixture in the formation of 2*n* eggs in a potato diploid clone. Theor. Appl. Genet..

[180] Correll D.S. (1962). The Potato and Its Wild Relatives: Section Tuberarium of the Genus Solanum.

[181] Dodds K.S., Correll D.S. (1962). Classification of cultivated potatoes. The Potato and Its Wild Relatives.

[182] Ellis D., Salas A., Chavez O., Gomez R., Anglin N., Campos H., Ortiz O. (2020). Ex situ conservation of potato [*Solanum* section Petota (Solanaceae)] genetic resources in genebanks. The Potato Crop: Its Agricultural, Nutritional and Social Contribution to Humankind.

[183] Nahirñak V., Almasia N.I., González M.N., Massa G.A., Oneto C.A.D., Feingold S.E., Hopp H.E., Rovere C.V. (2022). State of the Art of Genetic Engineering in Potato: From the First Report to Its Future Potential. Front. Plant Sci..

[184] Tiwari J.K., Challam C., Chakrabarti S.K., Feingold S.E. (2019). Climate Smart Potato: An integrated breeding, genomics and phenomics approach. Genomic Designing of Climate Smart Vegetable Crops.

[185] Raman R. (2017). The impact of Genetically Modified (GM) crops in modern agriculture: A review. GM Crops Food.

[186] González M.N., Massa G.A., Andersson M., Décima Oneto C.A., Turesson H., Storani L., Olsson N., Fält A.-S., Hofvander P., Feingold S.E. (2021). Comparative potato genome editing: *Agrobacterium tumefaciens*-mediated transformation and protoplasts transfection delivery of CRISPR/Cas9 components directed to *StPPO2* gene. Plant Cell Tissue Organ Cult..

[187] Zhu H., Li C., Gao C. (2020). Applications of CRISPR–Cas in agriculture and plant biotechnology. Nat. Rev. Mol. Cell Biol..

[188] Touzdjian Pinheiro Kohlrausch Távora F., de Assis dos Santos Diniz F., de Moraes Rêgo-Machado C., Chagas Freitas N., Barbosa Monteiro Arraes F., Chumbinho de Andrade E., Furtado L.L., Osiro K.O., Lima de Sousa N., Cardoso T.B. (2022). CRISPR/Cas- and Topical RNAi-Based Technologies for Crop Management and Improvement: Reviewing the Risk Assessment and Challenges Towards a More Sustainable Agriculture. Front. Bioeng. Biotechnol..

[189] Gonzalez M.N., Messa G.A., Andersson M., Turesson H., Olsson N., Falt A.-S., Storani L., Oneto C.A.D., Hofvander P., Feingold S.E. (2020). Reduced Enzymatic Browning in Potato Tubers by Specific Editing of a Polyphenol Oxidase Gene via Ribonucleoprotein Complexes Delivery of the CRISPR/Cas9 System. Front. Plant Sci..

[190] Tiwari J.K., Buckseth T., Challam C., Zinta R., Bhatia N., Dalamu D., Naik S., Poonia A.K., Singh R.K., Luthra S.K. (2022). CRISPR/Cas genome editing in potato: Current status and future perspectives. Front. Genet..

[191] Andersson M., Turesson H., Nicolia A., Fält A.S., Samuelsson M., Hofvander P. (2017). Efficient targeted multiallelic mutagenesis in tetraploid potato (*Solanum tuberosum*) by transient CRISPR-Cas9 expression in protoplasts. Plant Cell Rep..

[192] Ly D.N.P., Iqbal S., Fosu-Nyarko J., Milroy S., Jones M.G.K. (2023). Multiplex CRISPR-Cas9 Gene-Editing Can Deliver Potato Cultivars with Reduced Browning and Acrylamide. Plants.

[193] Kieu N.P., Lenman M., Wang E.S., Petersen B.L., Andreasson E. (2021). Mutations introduced in susceptibility genes through CRISPR/Cas9 genome editing confer increased late blight resistance in potatoes. Sci. Rep..

[194] Zhan X., Zhang F., Zhong Z., Chen R., Wang Y., Chang L., Bock R., Nie B., Zhang J. (2019). Generation of virus-resistant potato plants by RNA genome targeting. Plant Biotechnol. J..

[195] Massa G.A., Décima Oneto C.A., González M.N., Sucar S., Nadakuduti S.S., Arizmendi A., Poulsen Hornum A., Douches D., Feingold S.E. (2023). Papa cv. Atlantic Tolerante al Endulzamiento Inducido por frío Desarrollada por Edición Génica via CRISPR/Cas9. XIV Simposio REDBIO Argentina. https://www.redbioargentina.org.ar/simposio-2023/.

[196] Zsogon A., Cermak T., Naves E.R., Notini M.M., Edel K.H., Weinl S., Freschi L., Voytas D.F., Kudla J., Peres L.E.P. (2018). *De novo* domestication of wild tomato using genome editing. Nat. Biotechnol..

[197] Debernardi J.M., Tricoli D.M., Ercoli M.F., Hayta S., Ronald P., Palatnik J.F., Dubcovsky J. (2020). A GRF–GIF chimeric protein improves the regeneration efficiency of transgenic plants. Nat. Biotechnol..

